# Stereoselective virtual screening of the ZINC database using atom pair 3D-fingerprints

**DOI:** 10.1186/s13321-014-0051-5

**Published:** 2015-02-10

**Authors:** Mahendra Awale, Xian Jin, Jean-Louis Reymond

**Affiliations:** Department of Chemistry and Biochemistry, University of Berne, Freiestrasse 3, 3012 Berne, Switzerland

**Keywords:** Virtual screening, Chemical space, Databases, Fingerprints, Atom pairs, Molecular shape, Pharmacophores, Stereoselectivity

## Abstract

**Background:**

Tools to explore large compound databases in search for analogs of query molecules provide a strategically important support in drug discovery to help identify available analogs of any given reference or hit compound by ligand based virtual screening (LBVS). We recently showed that large databases can be formatted for very fast searching with various 2D-fingerprints using the city-block distance as similarity measure, in particular a 2D-atom pair fingerprint (APfp) and the related category extended atom pair fingerprint (Xfp) which efficiently encode molecular shape and pharmacophores, but do not perceive stereochemistry. Here we investigated related 3D-atom pair fingerprints to enable rapid stereoselective searches in the ZINC database (23.2 million 3D structures).

**Results:**

Molecular fingerprints counting atom pairs at increasing through-space distance intervals were designed using either all atoms (16-bit 3DAPfp) or different atom categories (80-bit 3DXfp). These 3D-fingerprints retrieved molecular shape and pharmacophore analogs (defined by OpenEye ROCS scoring functions) of 110,000 compounds from the Cambridge Structural Database with equal or better accuracy than the 2D-fingerprints APfp and Xfp, and showed comparable performance in recovering actives from decoys in the DUD database. LBVS by 3DXfp or 3DAPfp similarity was stereoselective and gave very different analogs when starting from different diastereomers of the same chiral drug. Results were also different from LBVS with the parent 2D-fingerprints Xfp or APfp. 3D- and 2D-fingerprints also gave very different results in LBVS of folded molecules where through-space distances between atom pairs are much shorter than topological distances.

**Conclusions:**

3DAPfp and 3DXfp are suitable for stereoselective searches for shape and pharmacophore analogs of query molecules in large databases. Web-browsers for searching ZINC by 3DAPfp and 3DXfp similarity are accessible at www.gdb.unibe.ch and should provide useful assistance to drug discovery projects.

**Graphical abstract:**
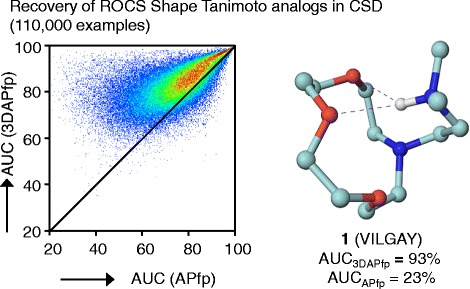
Atom pair fingerprints based on through-space distances (3DAPfp) provide better shape encoding than atom pair fingerprints based on topological distances (APfp) as measured by the recovery of ROCS shape analogs by fp similarity.

**Electronic supplementary material:**

The online version of this article (doi:10.1186/s13321-014-0051-5) contains supplementary material, which is available to authorized users.

## Background

Tools to explore large compound databases in search for analogs of query molecules provide a strategically important support for drug discovery and development projects to help identify available analogs of any given reference or hit compound by ligand based virtual screening (LBVS) [1-3]. While public compound databases such as ChEMBL [4] or ZINC [5] offer similarity searching on their websites, options are limited to a single type of 2D-substructure similarity comparisons, and performance is limited in terms of speed and number of analogs retrieved. Recently we reported a series of interactive database browsers, accessible at www.gdb.unibe.ch, allowing molecular fingerprint [6] based LBVS within seconds in very large databases of millions of compounds such as ZINC (13.2 M commercially available drug-like molecules), PubChem (53.2 M structures collected from public sources), [7,8] or the much larger Chemical Universe Databases GDB-11 (26.4 M), GDB-13 (977 M) and GDB-17 (166.4 G) enumerating all possible organic molecules following simple rules of chemical stability and synthetic feasibility up to 11, 13 and 17 atoms [9-13]. Fast LBVS was made possible by using the sum of fingerprint bit values as hash function and the city-block distance as fingerprint similarity measure, [14] an approach applicable to scalar fingerprints such as MQN (Molecular Quantum Numbers) [7] and SMIfp (SMILES fingerprint), [12] and to binary fingerprints such as the daylight type substructure fingerprint Sfp [15] and the extended connectivity fingerprint ECFP4 [16].

Due to the importance of 3D-molecular shape and pharmacophores in determining the bioactivity [17-25] and clinical success of small molecule drugs, [26] we recently expanded our city-block distance based search algorithm to the topological atom pair fingerprints APfp (20-bit atom pair fingerprint, all heavy atoms without categories) and Xfp (55-bit category extended atom pair fingerprint), which count the number of atom pairs at increasing topological distance, counted in bonds through the shortest path, following a concept originally reported by Carhart *et al*. [27] We showed that these fingerprints encode 3D-features of molecules in various enrichment studies for 3D-shape, 3D-pharmacophore, and bioactive analogs [28].

APfp and Xfp were computed from the 2D-structure only. Considering that the 3D-structure of molecules is now available in several large databases such as the Cambridge Structural Database (CSD, experimental X-ray crystal structure) or the collated catalogs of all commercial compounds (ZINC, predicted 3D-structures), it should also be possible to compute a related 3D-atom pair fingerprint considering through-space rather than topological distances between atoms and subsequently organize large databases for fast LBVS. Such 3D-fingerprints should represent the actual 3D-shape more closely than 2D-fingerprints, and enable stereoselective LBVS by distinguishing between different conformers and stereoisomers of the same molecule, which is not possible with 2D-fingerprints.

Sheridan *et al*. reported a 3D-atom pair fingerprint designed in direct extension of Cahart’s 2D atom pair fingerprint, counting all same-category pairs and cross-category pairs in different fingerprint bits using both Carhart’s original atom categories (atomic number, the number of π-electrons, number of non-hydrogen neighbors) and “binding property” categories (cation, anion, H-bond donor, H-bond acceptor, polar, hydrophobic, other) [29]. Sheridan’s approach resulted in a detailed pharmacophore fingerprint with good performance in 3D-similarity searches as exemplified in a database containing 30,000 molecules with an average of 10 calculated conformers per molecule. However the number of bits in Sheridan’s fingerprint was too large to be compatible with our rapid search algorithm for millions of molecules, therefore we set out to design a comparable but simpler 3D-atom pair fingerprint. Herein we report two new 3D-atom pair fingerprints closely related to our recently reported 2D-atom pair fingerprints in form of an “all atom” fingerprint treating all heavy atom equally (16-bit 3DAPfp), and a category extended fingerprint (80-bit 3DXfp) considering hydrophobic atoms (Hyb), H-bond donors (HBD), H-bond acceptors (HBA) and planar (sp^2^) as categories, and HBD-HBA as the only cross-pair. The fingerprints were evaluated in various LBVS studies in comparison with PMIfp (principal moments of inertia scaled to molecular weight collected in a scalar fingerprint), [17] USR (Ultrafast Shape Recognition) and USRCAT (atom category specific version of USR) [30,31] as examples of other types of 3D-fingerprints, [32-37] their parent 2D-fingerprints APfp and Xfp, and in selected cases MQN and Sfp. Fingerprints used in the present study are summarized in Table 1.

**Table 1:** **Fingerprints used in this study**

**Fingerprint**	**Feature perceived**	**Description** ^**a)**^	**Ref.**
3DAPfp	Shape	16-bit scalar 3D-fp, each bit is the sum of atom pair gaussian function values sampled at 16 different through-space distances between 1 and 20 Å, normalized to HAC^1.5^	^b)^
3DXfp	Pharmacophore	80-bit scalar 3D-fp, equivalent to 3DAPfp extended to 5 categories: Hyb, HBA, HBD, sp2, and cross-pair HBA-HBD	^b)^
R3DAPfp	Shape	40-bit scalar 3D-fp, each bit counts the number of atom pairs within the corresponding 0.5 Å through-space distance interval between 0 and 20 Å, normalized to HAC (R = regular binning)	^b)^
R3DXfp	Pharmacophore	200-bit scalar fp, category extended version of R3DAPfp	^b)^
APfp	Shape	20-bit scalar 2D-fp, each bit counts the number of atom pairs at one particular topological distance between 1 and 20 bonds, normalized to HAC	[28]
Xfp	Pharmacophore	55-bit scalar 2D-fp, category extended version of APfp	[28]
PMIfp	Shape	3-bit scalar 3D-fp, measures the principal moments of inertia scaled to molecular weight	[17]
USR	Shape	12-bit scalar 3D-fp, represents euclidean distance distributions calculated with respect to four chosen reference points by three statistical moments: average, standard deviation and kurtosis	[30]
USRCAT	Pharmacophore	60-bit scalar 3D-fp, version of USR extended with categories: All atoms, Hyb, HBA, HBD, aromatic atoms	[31]
MQN	Composition	42-bit scalar 2D-fp, counts 42 Molecular Quantum Numbers (MQN) counting atom types, bond types, polar groups and topologies	[7,8]
Sfp	Substructure	1024-bit binary 2D-fp, perceives the presence of substructures	[15]

^a)^3D-fp: fingerprint computed from the 3D-structure of a molecule. 2D-fp: fingerprint computed from the 2D-structure of the molecule. HAC = heavy atom count, all non-hydrogen atoms. Hyb = hydrophobic atoms, HBA = Hydrogen bond acceptor atoms, HBD = Hydrogen bond donor atoms, sp2 = planar, unsaturated atoms, HBA-HBD = HBA HBD cross-pair. ^b)^This work.

In a first study 3D-shape and pharmacophore analogs of 110,000 molecules from the Cambridge Structural Database (CSD) were defined using the Rapid Overlay of Chemical Structures (ROCS) shape similarity functions ROCS shape Tanimoto (shape only), ROCS Color Tanimoto (pharmacophore only), and ROCS Comboscore (combined shape and pharmacophore) [18,38,39]. Fingerprint based LBVS for these analogs showed that the very compact, 16-bit shape-only fingerprint 3DAPfp performed best among all fingerprints for recovering Shape and Comboscore analogs. 3DAPfp performed better than its 2D parent fingerprint APfp, in particular with molecules presenting a folded conformation in their crystal structure. On the other hand 3DXfp performed best for recovering pharmacophore (ROCS color) analogs from CSD. In a second study recovering actives in the directory of useful decoys (DUD), a broadly accepted method to benchmark virtual screening methods, [40-44] 3DXfp again performed better than 3DAPfp, yet showed results comparable to its parent 2D-fingerprint Xfp, an effect which might be related to the very 2-dimensional nature of the molecules in DUD and ZINC.

Remarkably, the 3D-fingeprints were stereoselective and produced significant differences between conformers and stereoisomers of the same molecule compared to different molecules of similar size. A third study was therefore performed in which the 3D-fingerprints were used for LBVS starting from different diastereomers of chiral drugs. Both 3DXfp and 3DAPfp gave very different nearest neighbors from different diastereomers, which were also different from the nearest neighbours obtained by the parent 2D-fingerprint search with Xfp or APfp, highlighting the impact of stereochemistry on LBVS. 3D-fingerprints also returned different nearest neighbors compared to 2D-fingerprints when searching for analogs of folded molecules identified as bound ligands in the Protein Databank. 3DAPfp and 3DXfp were used to design web-browsers for the 23.2 million 3D-structures in the ZINC database, which is freely available at www.gdb.unibe.ch. Stereoselective LBVS of 3D-structures in ZINC should provide useful assistance for drug discovery projects.

## Results and discussion

### Fingerprint design and optimization

**Figure 1:**
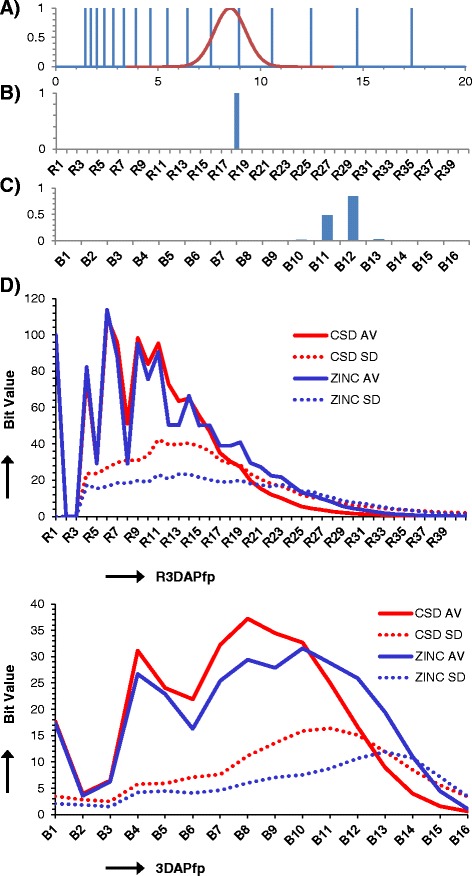
**3D-atom pair fingerprint design. A-C**. Distance sampling for 3D-atom pair fingerprints illustrated for atom-pair distance of 8.51 Å. **A**. A gaussian curve is drawn (red) with its maximum centred at atom-pair distance of 8.51 Å and width as 18% of atom-pair distance. The gaussian is then sampled at 16 distance values B1-B16 (blue vertical bars): 1.45, 1.71, 2.02, 2.38, 2.81, 3.32, 3.91, 4.62, 5.45, 6.43, 7.59, 8.96, 10.57, 12.47, 14.71 and 17.36 Å (16 bit values at d_n+1_ = d_n_ × 1.18) **B**. Regular Binning: the atom-pair distance of 8.51 Å produces an increment of 1 in the R18 bin covering the range of 8.5-9 Å. **C.** Bit values B1-B16 for the atom pair at 8.51 Å from the gaussian/exponential sampling principle in A. **D**. Average bit value and standard deviation (SD) of R3DAPfp and 3DAPfp of all molecules from the Cambridge structural database (CSD, 110 000 molecules) and ZINC (23.2 M molecules).

The 3D-fingerprints were designed in direct analogy to our recently reported 2D atom pair fingerprints, with a simple version tailored for shape similarity with all heavy atoms treated equally (3DAPfp), and an atom category extended version (3DXfp) tailored for pharmacophore similarity, considering hydrophobic atoms (Hyb), H-bond donors (HBD), H-bond acceptors (HBA), planar atoms (sp^2^), and the HBD-HBA cross-pair as categories. In contrast to 2D-fingerprints for which distance bins are automatically defined by the topological distance counted in number of bonds through the shortest path, 3D-fingerprints require a binning principle for the through-space distance to assign atom pairs to distance bins. Following an approach similar to that of Sheridan et al., [29] each through-space atom-pair distance was converted to a gaussian function with its maximum value at the atom pair distance and a width of 18% of the atom pair distance, and the function was sampled at 16 values between 1.45 Å and 17.36 Å, each interval between sampling values being 1.18 times broader than the preceding interval (16-bit 3DAPfp and 80-bit 3DXfp). The atom pair bit value increments were summed, and the sum values normalized to HAC^1.5^, which reduced sensitivity to molecular size. This gaussian/exponential sampling principle allowed for a certain degree of fuzziness in the shape perception at large distances while reducing the dimensionality of the fingerprint. To test if this concept was useful, two additional 3D-fingerprints were created by simply binning the distance at regular 0.5 Å intervals up to 20 Å and assigning each atom pair to a single bit, normalizing bit values to the heavy atom count (regular binning: 40-bit R3DAPfp and 200-bit R3DXfp). For each of the four fingerprints (3DAPfp, 3DXPfp, R3DAPfp and R3DXfp), the bit values were expressed in percent and rounded to the integer value. The fingerprint design and bit-value profiles of R3DAPfp and 3DAPfp for the reference databases CSD and ZINC are illustrated in Figure 1.

The performance of the 3D-atom pair fingerprints 3DAPfp, R3DAPfp, 3DXfp and R3DXfp was evaluated in analog enrichment studies discussed below. In the course of these studies, parameter variations were examined to challenge the design of 3DAPfp and 3DXfp, which confirmed that the selected width of the atom pair gaussian (18% of atom pair distance) and the multiplication factor between successive sampling intervals (1.18) were optimal. For the regular binning fingerprints R3DAPfp and R3DXfp optimal results were obtained using 0.5 Å bin width, with broader but fewer bins giving slightly better results for recovering 3D-shape and pharmacophore analogs, and narrower but more numerous bins giving slightly better results in the DUD enrichment studies (Additional file 1: Figures S1-S3).

### LBVS in the Cambridge structural database

**Figure 2:**
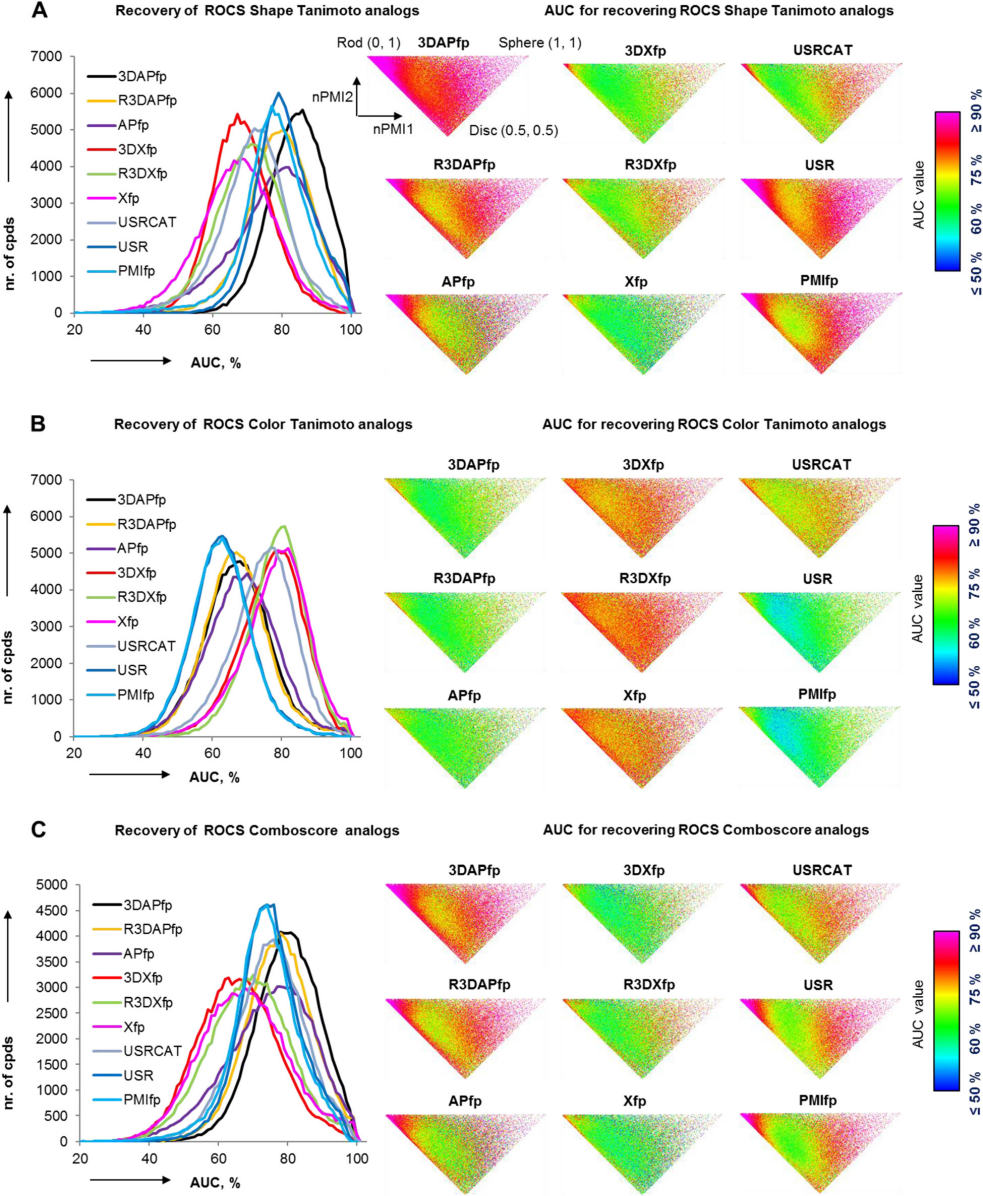
**Recovery statistics of 100 closest analogs of CSD molecules according to ROCS Shape Tanimoto (A), Color Tanimoto (B) and ComboScore (C), by LBVS using various fingerprints, for each of the 110,000 molecules in CSD from their size-constrained subsets (all CSD molecules within HAC = query ± 2).** For each of the three cases (**A-C**), the frequency histogram of AUC values for various fingerprints is shown on left, and the average AUC value as a function of position in the shape triangle for various fingerprints is shown on right. The shape triangle results from plotting the normalized moment of inertia of molecules and distinguishes rod-like, disc-like and sphere-like shapes. Continuous color scale: AUC ≤ 50%: blue, 58%: cyan, 66%: green, 75%: yellow, 80%: red, ≥ 90%: magenta. See also Additional file 1: Figure S1 and S2 in the SI for data showing recovery statistics for different variants of 3DAPfp, 3DXfp, R3DAPfp and R3DXfp.

LBVS for 3D-shape and pharmacophore analogs using the various fingerprints was tested for 110,000 organic molecules up to 50 atoms from the Cambridge Structural Database CSD, which reports experimentally determined 3D coordinates covering a broad range of molecular shapes as measured by the normalized principal moment of inertia (nPMI) triangle, [17] including significant coverage of disk-like and spherical shapes. For each of the 110,000 CSD molecules, three series of “actives” were defined as the 100 closest shape, pharmacophore, or shape + pharmacophore analogs, which were the 100 highest scoring CSD compounds according to one of the following three scoring functions: ROCS (Rapid Overlay of Chemical Structures) shape Tanimoto (3D-shape), ROCS Color Tanimoto (3D-pharmacophore), and ROCS Comboscore (combined 3D-shape and 3D-pharmacophore) [18,38]. The receiver operator characteristics (ROC) curves were then computed for each of the 110,000 CSD compounds for retrieving each for the three series of 100 “actives” (3D-shape and pharmacophore analogs) from a size-constrained subset of CSD (containing all molecules of size HAC ± 2) by LBVS using each of the different fingerprints (Figure 2).

Atom-pair fingerprints performed significantly better than USR, USRCAT and PMIfp in these comparisons, probably reflecting the more detailed encoding of molecular shape through atom pair counts compared to the more global shape parameters encoded in USR, USRCAT and PMIfp. The very compact 16-bit shape fingerprint 3DAPfp stood out by its high LBVS performance for ROCS shape analogs, which was higher than for R3DAPfp and the parent 2-dimensional APfp, showing that the gaussian/exponential binning principle used for 3DAPfp contributed to a better molecular shape perception (Figure 2A). The atom category extended fingerprint 3DXfp showed higher performance than 3DAPfp for recovery of ROCS Color Tanimoto analogs, in line with the fact that ROCS Color primarily encodes pharmacophores. However in this case results with 3DXfp were comparable to R3DXfp and the parent 2D-fingerprint Xfp independent of any position in the shape triangle (Figure 2B). Recovery of ROCS Comboscore analogs was most efficient using 3DAPfp, showing that this ROCS scoring function, which combines shape and pharmacophores, is dominated by molecular shape (Figure 2C).

**Figure 3:**
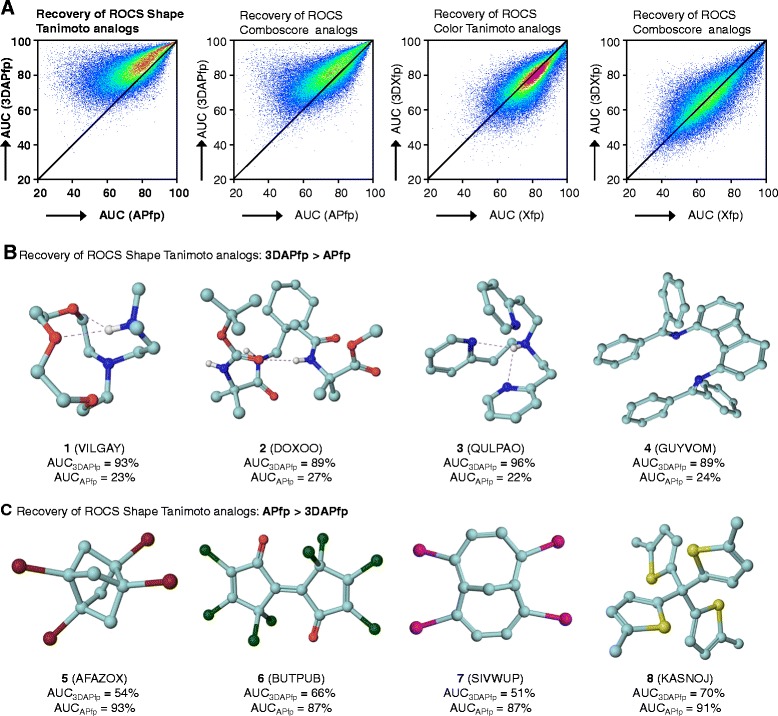
**Recovery of ROCS analogs in CSD using 3D and 2D fingerprints. A**. AUC values with 3DAPfp or 3DXfp (y-axis) vs. AUC values with APfp or Xfp (x-axis). The scatter plots are coloured according to compounds/pixel: Red = ≥25, Yellow = 19, Green = 12, Cyan = 6 and Blue = 1. **B**. Examples for which recovery of ROCS Shape Tanimoto analogs is better with 3DAPfp than with APfp. **C**. Examples for which recovery is better with APfp than with 3DAPfp.

Analysis of the AUC values for recovery of ROCS analogs of individual CSD compounds using 3D vs. the corresponding 2D fingerprint further illustrated the generally superior performance of 3DAPfp vs. APfp, and the comparable performance of 3DXfp and Xfp (Figure 3A). For cases where the AUC values were higher for 3DAPfp than for APfp such as compounds **1**–**4**, a folded conformation was observed in the crystal structure. In such folded structures topological distances overestimate the actual through-space distances separating atom pairs, explaining the lower performance of the 2D-fingerprint. The folded conformation was caused by intramolecular H-bonds in the case of **1**–**3** and a π-stack effect in compound **4** (Figure 3B). On the other hand, the 2D-fingerprint APfp performed better than 3DAPfp in a significant number of cases, in particular for molecules with a large number of sulfur and halogen atoms as for **5**–**8** (Figure 3C). This effect is difficult to rationalize because it occurs independent of molecular shape in both planar (*e.g*. **6** and **7**) and spherical (*e.g*. **5** and **8**) molecules.

### DUD enrichment studies

**Figure 4:**
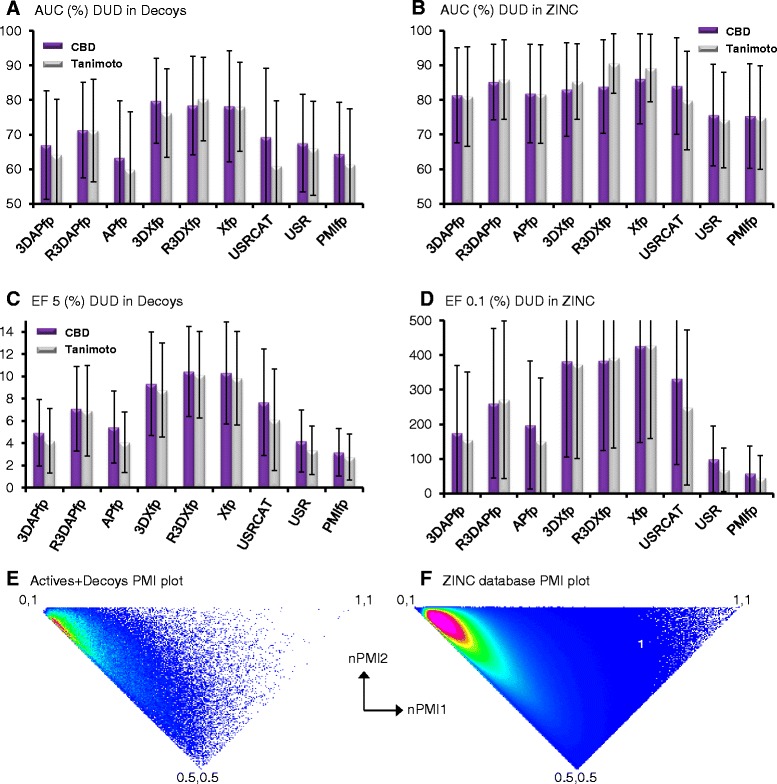
**Recovery of DUD actives using various fingerprints. (A)** Average AUC values and (**C**) enrichment factors at 5% (EF5%) for recovery of 40 sets of actives in directory useful decoys (DUD) from the corresponding decoys set by various fingerprints, using CBD _fingerprint_ (violet bars) and *T*_fingerprint_ (grey bars) as scoring functions. (**B**) AUC values and (**D**) EF0.1% values for recovery of DUD actives from the entire ZINC database. **(E)** Occupancy heat map of the molecular shape triangle by DUD actives and decoys (128,352 cpds, blue ≤ 2 cpd/pixel to magenta ≥ 150 cpds/pixel) and (**F**) by the entire ZINC database (23.2 M cpds, blue ≤ 50 cpd/pixel to magenta ≥ 10000 cpds/pixel). See Additional file 1: Table S1-S8 for detailed AUC and EF values and Additional file 1: Figure S4-S7 for ROC curves.

The recovery of DUD actives from decoys and from the entire ZINC database was investigated as a second test for fingerprint performance [40-44]. For each DUD active set the molecule closest to all other actives in the set in the corresponding fingerprint space was used as reference molecule for the recovery study. LBVS for recovering the other actives from this reference molecule gave comparable results using either the city-block distance or the Tanimoto coefficient as similarity measures (Figure 4A-D and Additional file 1: Figures S4-S7 and Tables S1-S8). 3DXfp, R3DXfp and Xfp stood out as the fingerprints showing the highest average AUC values (~80%) and enrichment factors at 5% coverage (first 1000–2000 cpds, EF5% = 8–10) for the recovery of actives from the corresponding decoys. The other fingerprints performed significantly lower (AUC ~ 60–70 %, EF5% ~ 2–8). The recovery of DUD actives from the entire ZINC database was quite good with all fingerprints (average AUC ~ 80–90%) except USR and PMIfp (average AUC ~ 75%), however enrichment factors at 0.1% database coverage (first 23,200 cpds) were higher for pharmacophore fingerprints (3DXfp, R3DXfp, Xfp,USRCAT) than for shape only fingerprints.

The various 3D atom pair fingerprints readily retrieved scaffold-hopping analogs, which are compounds with high shape and pharmacophore similarity, similar bioactivity, but a low level of substructure similarity as measured by substructure similarity comparisons (Sfp) [45]. Examples of scaffold-hopping analogs among DUD actives retrieved by 3DXfp are shown in Additional file 1: Figure S8. Similar scaffold-hopping capabilities were reported previously with MQN, APfp and Xfp, and generally occur with fingerprints not taking detailed substructures into account.

It should be noted that most molecules in DUD and ZINC are rod-like or at best 2-dimensional with only very few 3D-shaped molecules (Figure 4E/F). The very low shape diversity in these databases might partly contribute to the similar LBVS performance of 3D and 2D methods with DUD also noted in previous literature reports [18,33,41,42,46-49].

### Stereoselective LBVS

A distinctive feature of 3D-scoring functions and fingerprints is their ability to distinguish between different stereoisomers and conformers of the same molecule. Indeed the 3D-fingerprints investigated here distinguished between various stereoisomers and conformers of the model cases 4,5-dihydroxy-octa-2,6-diyne (2 enantiomers and one *meso* form, 9 conformers), glucopyranose (32 possible diastereomeric hexopyranoses, 154 conformers) and arachidonic acid ((5*Z*,8*Z*,11*Z*,14*Z*)-5,8,11,14-eicosatetraenoic acid, 16 possible *E/Z* double bond isomers, 640 conformers). However they lacked chiral sense information and did not differentiate between mirror image conformers, a possibility offered by ROCS scoring functions computed from overlapping chiral 3D-structures (Additional file 1: Figure S9).

**Figure 5:**
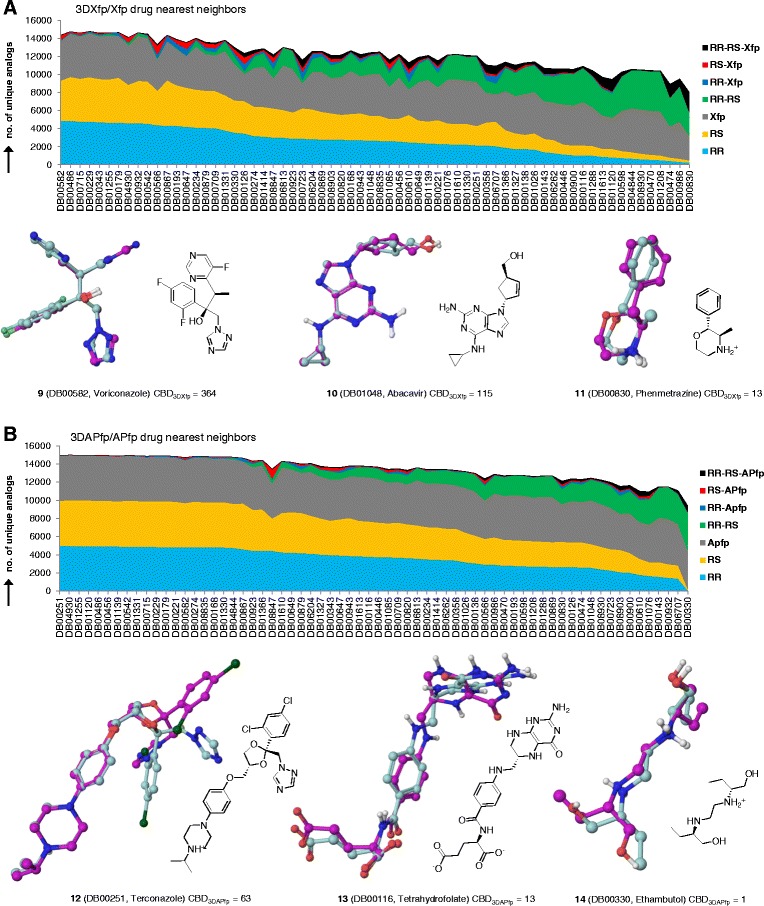
**LBVS in ZINC (23.2 M 3D-structures) for analogs of 66 marketed drugs with two asymmetric centers. A.** Cumulative area plot giving the number of unique compounds among 5000 3DXfp nearest neighbors of RR and RS diastereomers of each drug and 5000 Xfp nearest neighbors of the same drug as found only from RR (cyan), RS (yellow), Xfp (grey), RR and RS (green), RR and Xfp (blue), RS and Xfp (red), or in all three searches (black). The drugs (DrugBank code on x-axis) are sorted by decreasing number of RR-unique analogs. Examples of diastereomers of drugs are shown in overlayed magenta/cyan models of the energy minimized conformers used for LBVS. **B**. Same as **A** for the case of 3DAPfp and APfp as fingerprints.

To test if the stereoselectivity of 3D-fingerprints might influence LBVS, 66 marketed drugs with two stereocenters were identified in Drugbank, and the lowest energy conformer was generated using Omega for each of the two possible diastereomers RR and RS [50]. The 5000 3DXfp nearest neighbors in ZINC (23.2 M 3D-structures) of each diastereomer of the 66 drugs and the 5000 Xfp nearest neighbors of the corresponding 2D-structures in ZINC were then retrieved and assigned as exclusively found in one, two or three of the individual searches (Figure 5A). The same study was performed using 3DAPfp and APfp as fingerprints (Figure 5B). Approximately 25% of the searches delivered essentially completely different hits from the RR, RS and the 2D-fingerprint search. For example voriconazole (**9**) / terconazole (**12**) gave the most diastereoselective search results in the Xfp/APfp searches. In both cases the diastereomers presented large aromatic substituents in opposite relative orientation in space in the minimum energy 3D-conformation used for LBVS. The remaining drugs gave decreasingly stereoselective search results reflecting increasing 3D-shape similarity between the RR and RS diastereomers. For example abacavir (**10**) diastereomers only differed in the cyclopentene stereochemistry and ring conformation and shared 28% of their 3DXfp nearest neighbors, while tetrahydrofolate (**13**) diastereomers differed in the orientation of their biopterin ring and shared 12.5% of their 3DAPfp nearest neighbors. At the end of the list the diastereomers of phenmetrazine (**11**) were almost superimposable and shared 89% of their 3DXfp nearest neighbors. Similarly ethambutol (**14**) diastereomers, which are identical in 3D-shape when ignoring atom types, shared 94% of their 3DAPfp neighbors. In all cases the 3D and 2D-fingerprint searches were almost entirely different, illustrating the different shape perception from through-space versus topological distances. The very different nearest neighbors of diastereomeric drugs confirmed the ability of the 3D-atom pair fingerprints to represent stereochemistry and conformation and underscored their importance in LBVS from 3D-structures.

### LBVS with folded molecules

3D-fingerprints should behave differently from 2D-fingerprints in LBVS with folded molecules where through-space distances determining molecular shape are much shorter than topological distances (*e.g.***1**–**4** Figure 3). To illustrate this point 10 ligands bound to their target protein in a folded conformation were identified by searching the Protein Databank for small molecules with very low correlation coefficient between through-space distance between atom pairs in the 3D-structure of the conformer and the corresponding atom pair topological distances in the parent 2D-structure (Additional file 1: Figure S10). In all 10 cases similarly folded conformations were generated from the Open Eye Omega 3D-builder (with which the 3D-structures in ZINC were computed), implying that folding was intrinsic and not induced by protein binding.

**Figure 6:**
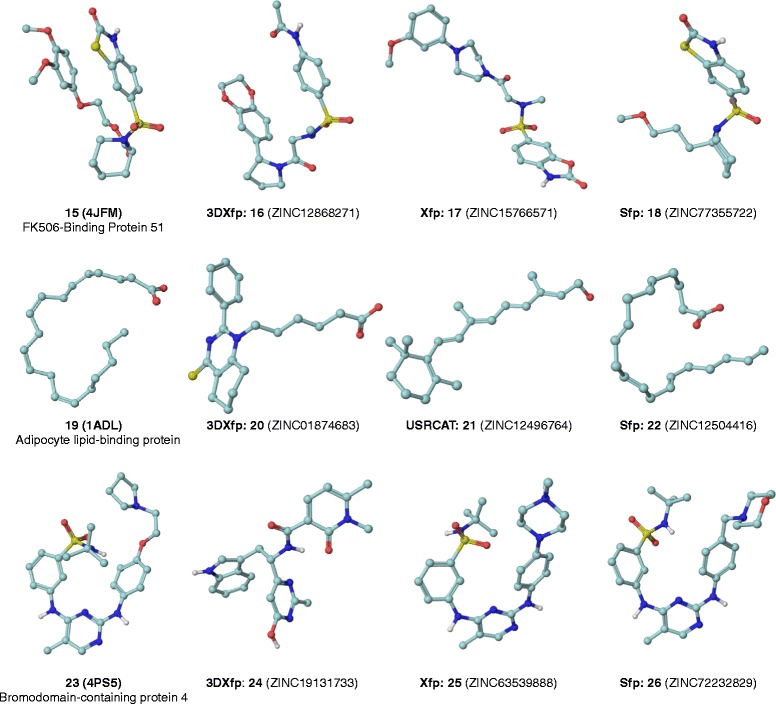
**Example of protein bound folded molecules and closest analogs (rank 1 or 2) identified in ZINC by 3D- and 2D-fingerprint similarity.** The pdb-entry code of the protein-ligand complexes or the ZINC ID number are given in parentheses for each compound. See also Additional file 1: Figures S10 and S11.

The 3D-shape and pharmacophore similarity of ZINC nearest neighbors of these 10 folded compounds in the various fingerprint spaces was generally very low (ROCS scores, Additional file 1: Figure S11) indicating that very few good analogs were available in ZINC. Nevertheless the closest neighbors illustrated the differences between LBVS using 3D- and 2D-fingerprints (Figure 6). In the case of the FKBP ligand **15** featuring a pair of π-stacked aromatic groups bound via a pipecolic amide sulfonamide linker in a turn conformation, molecule **16** retrieved as the first hit in the 3DXfp nearest neighbor search presented a pair of aromatic rings with comparable substitution and in a similar orientation, a feature which was lacking in compound **17** ranked first by Xfp and in **18** ranked first by Sfp. In the case of arachidonic acid **19** bound to the adipocyte lipid-binding protein, 3DXfp proposed as second rank analog hexanoic acid **20** with a hydrophobic and bulky tricyclic aromatic group at position 6 mimicking the folded aliphatic chain of **19**. USRCAT interestingly proposed retinol **21** as closest analog. Sfp by contrast retrieved simple straight-chain unsaturated carboxylic acids such as the all-*trans* eicosatetraenoic acid **22** at rank 2, a trend which was also present in Xfp analogs where topological distance perception favoured linear chain analogs, nevertheless many of these straight chain analogs presented a similarly folded conformation. In the case of bromodomain inhibitor **23** the closest neighbor in 3DXfp space was the unusual scaffold-hopping analog **24**. Xfp and Sfp nearest neighbors by contrast were standard substructure analogs such as **25** (rank 2) and **26** (rank 2) presenting the same folded conformation. The folded conformation of analogs **22**, **25** and **26** retrieved by 2D-fingerprints illustrates that conformational preferences including folding are often enforced by the 2D-structure and therefore indirectly perceived by 2D-fingerprints. Taken together, the data showed that 3D-fingerprints performed very differently from 2D-fingerprints when searching for analogs of folded molecules, in particular by pointing to analogs with very different scaffolds but realizing similar occupancy of 3D-space.

### 3DXfp and 3DAPfp browsers

**Figure 7:**
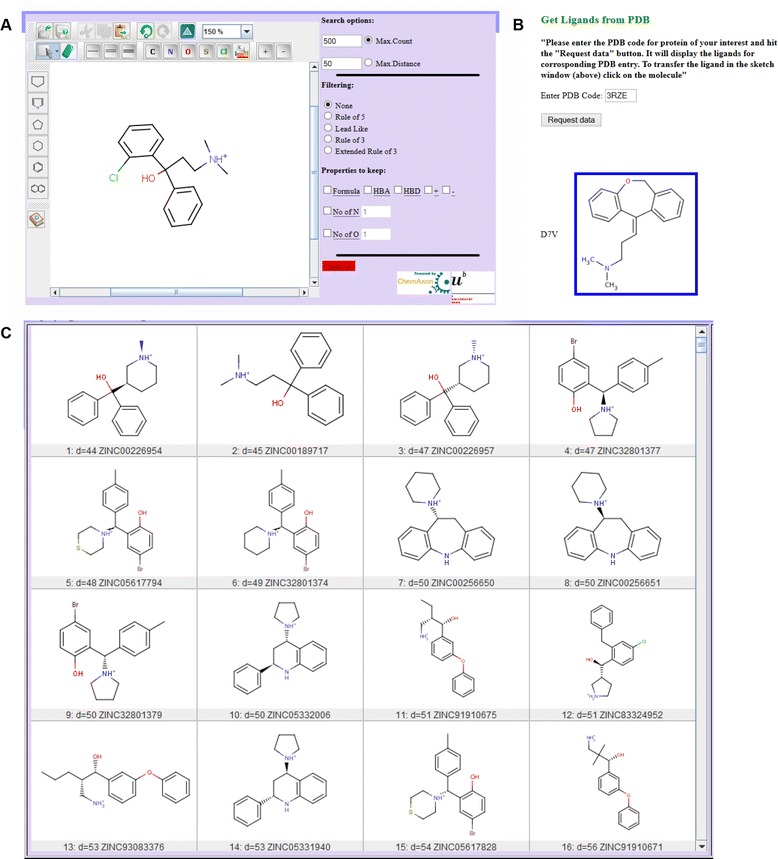
**Graphical user interface of the 3DXfp browser at** www.gdb.unibe.ch **with clofedanol (DrugBank ID: DB04837) as query example. A.** Molecule drawing window: the query molecule can be drawn or copy pasted as SMILES or SDF or MOL2 format. **B.** Alternative entry window for ligands from PDB with doxepin loaded from 3RZE, Histamine H1 receptor, as example. **C.** Result window displaying the 3DXfp nearest neighbors of Clofedanol.

The 3DAPfp and 3DXfp data computed for the ~ 23.2 M 3D-structures provided in the ZINC database were formatted for fast searching using a web-browser similar to those reported previously for other fingerprints, which allow retrieving city-block distance nearest neighbors of any given query molecule within a few seconds [9-12]. The 3DAPfp- and 3DXfp-browsers for ZINC are available online at www.gdb.unibe.ch. The search for 3DXfp-nearest neighbors of the drug Clofedanol in ZINC is shown to illustrate the user interface (Figure 7). The query molecule can be entered in the drawing window by drawing or by pasting the molecule in SMILES, sdf or MOL2 format (Figure 7A), or loaded directly from the pdb-entry of a known protein ligand complex (Figure 7B). If a structure is entered as SMILES without stereochemistry or 3D-structure one low energy stereoisomer and conformer is generated by the default options of the CORINA 3D-builder [51]. One can then search up to a preset number of CBD nearest neighbors or a preset CBD value. Additional search criteria to focus search results include compliance to Lipinski’s rule of five, [52] Oprea’s lead-likeness, [53] and Congreve’s rule of three and extended rule of three criteria, [54] locking the elemental formula (isomer search), the number of HBA, HBD, positive and negative charges, and the desired number of N or O atoms (Figure 7A). These options can be used to add pharmacophore criteria to the shape-only 3DAPfp search, and to enforce electrostatic charge information, which is not encoded in the fingerprints.

Search times for retrieving 1000 nearest neighbours with the browsers are approximately 16 ± 10 sec. for 3DAPfp and 43 ± 17 sec. for 3DXfp depending on molecule size and the availability of closely related analogs in ZINC, to which data transfer times via the internet connection must be added. The search results are limited to a maximum of 1000 molecules to avoid stalling of the internet browser. The search results are displayed as molecule matrix indicating for each molecule the city-block distance to the query and the ZINC ID number (Figure 7C). For each of the result molecules, a link option is available to visualize the data in the parent ZINC database. The interactive browsers provide a straightforward method to rapidly interrogate ZINC for 3D-shape and 3D-pharmacophore analogs of any molecule of interest.

## Conclusion

Extending on the work of Sheridan et al., [29] geometric atom pair fingerprints counting atom pairs for all heavy atoms or extended with atom categories at increasing through-space distances were designed considering either fuzzy atoms pairs binned into increasing distance intervals (3DAPfp and 3DXfp), or direct binning of the exact atom-pair distance in 0.5 Å distance intervals (R3DAPfp and R3DXfp). These 3D fingerprints were compared in LBVS performance with other 3D-fingerprints (PMIfp, USR and USRCAT), the corresponding topological atom pair fingerprints APfp and Xfp, and MQN and Sfp as reference 2D-fingerprints. LBVS performance was assessed in enrichment studies for ROCS Shape and pharmacophore analogs in CSD and in the recovery of actives in DUD from decoys and from ZINC. The data showed that 3DAPfp was the best fingerprint for representing 3D-shape as measured by the ROCS Shape Tanimoto and Comboscore scoring functions, in particular surpassing its parent 2D-fingerprint APfp. On the other hand 3DXfp surpassed 3DAPfp for LBVS of ROCS pharmacophore analogs and DUD actives, however its performance was comparable to its parent 2D-fingerprint Xfp.

LBVS with 3DXfp and 3DAPfp was stereoselective, leading to very different nearest neighbors from diastereomeric drugs as query molecules. LBVS results with 3DXfp and 3DAPfp were themselves different from nearest neighbors retrieved using the 2D-fingerprints Xfp and APfp. 3D-and 2D-fingerprints also retrieved substantially different molecules as nearest neighbors of folded molecules for which through-space distances between atom pairs are much shorter than topological distances. An interactive browser was assembled for searching through the 23.2 million 3D-structures in the ZINC database according to 3DAPfp and 3DXfp similarity, which is accessible at www.gdb.unibe.ch. Such web-browser for stereoselective LBVS of ZINC should provide useful assistance to drug discovery projects.

## Methods

### Databases

ZINC (https://docking.org/) and DUD (http://dud.docking.org/) databases were downloaded in SDF format from respective database websites. The 3D-structures in ZINC are lowest energy conformers (one conformer per molecule) calculated with Omega [50]. Cambridge Structural Database (CSD) was copied from a licensed CD to Dr. Jürg Hauser, University of Bern. All the calculations were performed on 3D structural information available in downloaded SDF files. Counter ions were removed and ionization state of molecules were adjusted to pH 7.4, using an in-house built java program utilizing Java Chemistry library (JChem) from ChemAxon, Ltd., as a starting point. In case of CSD, compounds up to 50 heavy atoms (~110 k) were considered in the presented study. If the compound was available in complex form, only one of the largest fragments was retained.

### 3D atom pair fingerprints

Computation of 3DAPfp, 3DXfp and all the other fingerprints were carried out using an in-house written java program utilizing various plugins of Java Chemistry library (JChem) from ChemAxon, Ltd., as a starting point.

The 40-bit R3DAPfp was constructed as follows: For each atom pair AB in the molecule, an increment of 1 was added in the bit of the 0.5 Å interval containing the atom pair distance d_AB_ between 0 and 20 Å. The summed bit-values were divided by HAC (heavy atom count), multiplied by 100, and rounded to the integer value. Rounding reduces the size of data for storage and has no significant influence on LBVS results [28]. For the 200-bit R3DXfp atoms were assigned to one of more of the following four categories: hydrophobic (Hyb), Hydrogen Bond Donor (HBD), Hydrogen Bond Acceptor (HBA), planar (sp2), and the R3DAPfp was computed within each of the four same-category pair (Hyb-Hyb, HBA-HBA, HBD-HBD, sp2-sp2) and for the HBA-HBD cross-pairs normalized to HBA.

The 16-bit 3DAPfp was constructed as follows: For each of the atom pair AB in the molecule, a gaussian function was generated centered at the atom pair distance d_AB_ with width of 0.18 × d_AB_, and the function was sampled at 1.45, 1.71, 2.02, 2.38, 2.81, 3.32, 3.91, 4.62, 5.45, 6.43, 7.59, 8.96, 10.57, 12.47, 14.71 and 17.36 Å (16 bit values at d_n+1_ = d_n_ × 1.18). For each of the 16 bits, values were summed across all atom pairs, the sum was divided by HAC^1.5^, multiplied by 100, and rounded to the integer value. For the 80bit 3DXfp the 3DAPfp was similarly computed within each of the atom type categories (see R3DXfp above).

### MQN and Sfp

MQN was calculated using the previously reported source code (freely available at www.gdb.unibe.ch) written in Java [7,12]. For the substructure fingerprint Sfp, a daylight type 1024-bit hash fingerprint with path length of 7 was computed using JChem library.

### PMIfp and triangular shape plot

PMIfp calculation were adopted from Sauer and Schwarz [17] and was written in Java as described previously [55].

### USR and USRCAT

Source code for the USR [30] (Ultra-fast Shape Recognition) fingerprint calculation was obtained from the Chemistry Development Tool Kit (CDK, http://sourceforge.net/projects/cdk/files/cdk/1.4.19/) and used to compute 12 dimensional USR (4*3 moments) shape fingerprint for the molecule.

Computation of USRCAT was facilitated by the python source code obtained from the https://bitbucket.org/aschreyer/usrcat/ website. Five atom pair categories namely: a) All atoms b) Hydrophobic c) Aromatic atoms d) HBA and e) HBD were created in USRCAT. Similar to the USR, moments were generated for each of the five categories which results in the 60 bit (12 × 5) USRCAT fingerprint.

## Additional file

Additional file 1:
**A supporting information pdf file is provided containing: Figure S1-S3 for fingerprint optimization data; Tables S1-S8 for AUC/EF Values and Figures S4-S7 for ROC curves for the DUD study; Figure S8 for examples of scaffold-hopping analogs; Figure S9 for stereoisomer and conformer comparisons of Diol, Glucose and Arachidonic acid; Figure S10 for correlation of topological and through-space distances for small molecules from PDB; Figure S11 for average ROCS similarity scores for 10,000 nearest neighbors of 10 folded compounds.**
