# Supplementary material for: Stereoselective virtual screening of the ZINC database using atom pair 3D-fingerprints
Source: J Cheminform. 2015 Feb 10;7:3. doi: 10.1186/s13321-014-0051-5 (PMC4352573; doi:10.1186/s13321-014-0051-5)
Supplement: Additional file 1: — A supporting information pdf file is provided containing: Figure S1-S3 for fingerprint optimization data; Tables S1-S8 for AUC/EF Values and Figures S4-S7 for ROC curves for the DUD study; Figure S8 for examples of scaffold-hopping analogs; Figure S9 for stereoisomer and conformer comparisons of Diol, Glucose and Arachidonic acid; Figure S10 for correlation of topological and through-space distances for small molecules from PDB; Figure S11 for average ROCS similarity scores for 10,000 nearest neighbors of 10 folded compounds. [file 13321_2014_51_MOESM1_ESM.pdf]

Supporting Information for:

Stereoselective Virtual Screening of the ZINC Database Using Atom Pair 3D-Fingerprints

Mahendra Awale, Xian Jin and Jean-Louis Reymond*

Department of Chemistry and Biochemistry, University of Berne, Freiestrasse 3, 3012 Berne Switzerland; e-mail: jean-louis.reymond@ioc.unibe.ch; FAX: +41 31 631 80 57

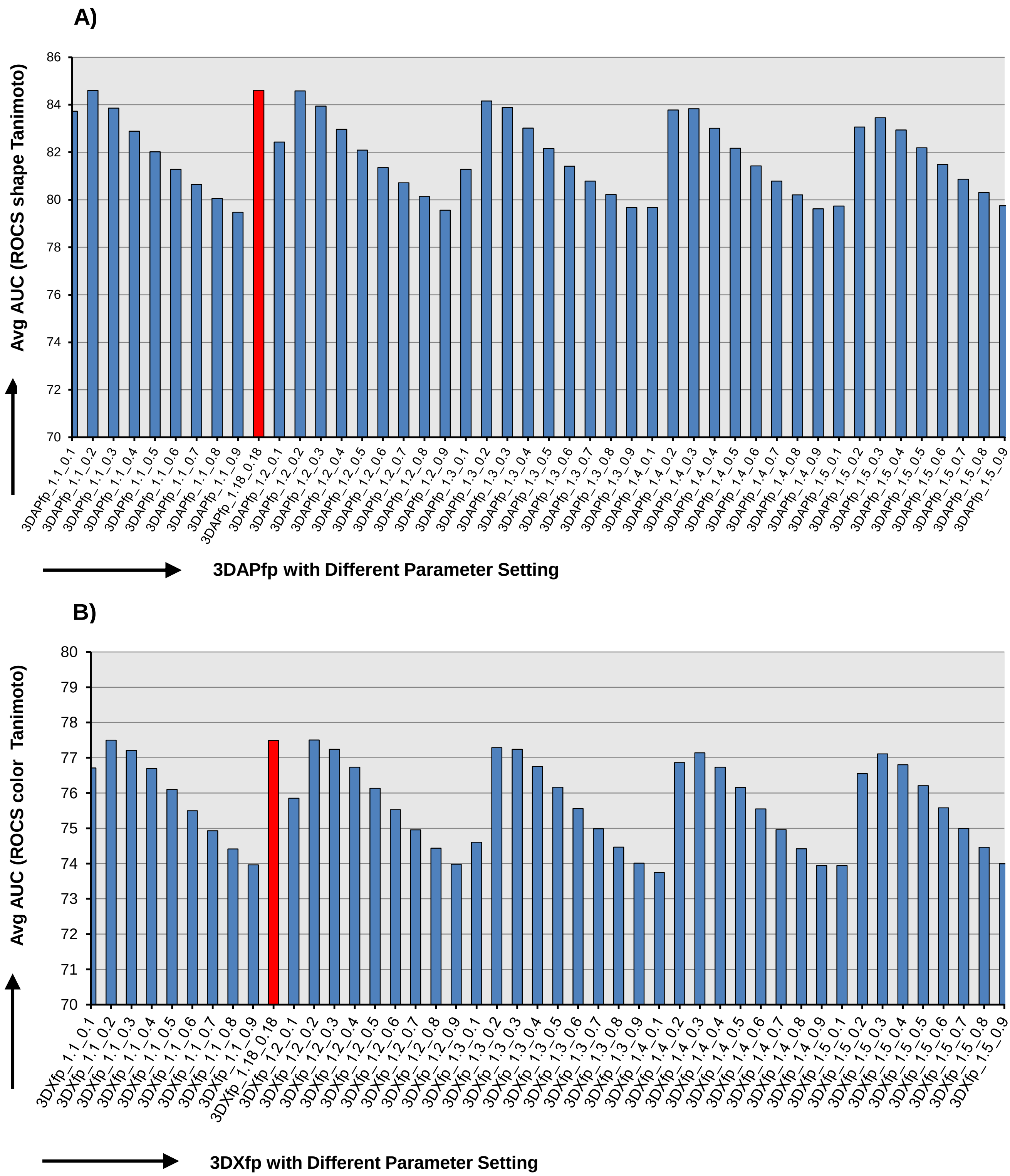

Figure S1. Optimization of 3DAPfp and 3DXfp parameters. Data is represented as average of Area under ROC curve for recovery of 100 nearest neighbors for each of the 145,000 molecules in CSD from their size-constrained subsets (all CSD molecules within HAC = query ± 2). A) Recovery of ROCS shape Tanimoto analogs and b) Recovery of ROCS Color Tanimoto analogs. 3DAPfp_X_Y/3DXfp_X_Y: X = bit width size factor in exponential binning of distance axis $d_{n+1} = d_n \times X$. Y = controls the width of Gaussian curve as Y × d_{AB} (where d_{AB} = atom pair distance).

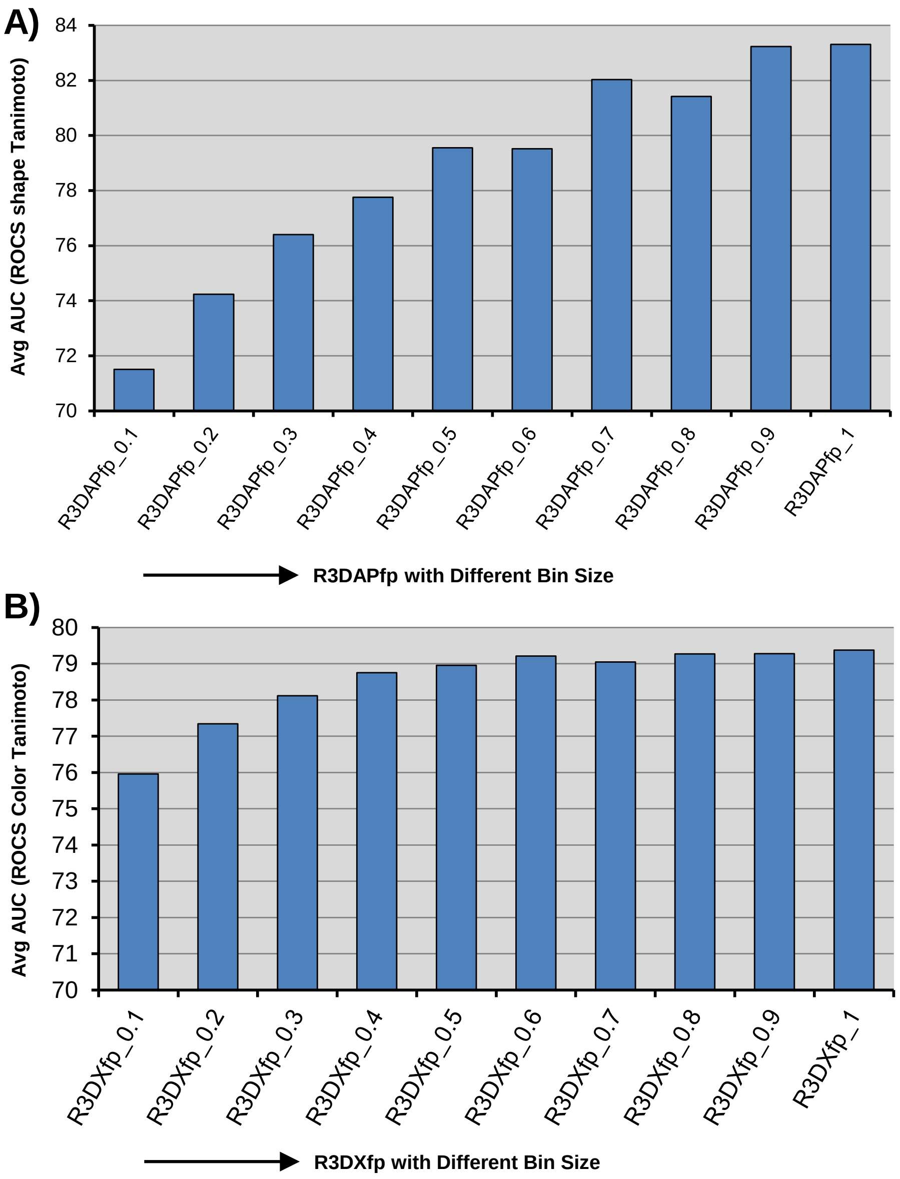

Figure S2. Performance of R3DAPfp and R3DXfp variants with different bin sizes (R3DAPfp_x or R3DXfp_x) used for fingerprint construction. Data is represented as average of Area under ROC curve for recovery of 100 nearest neighbors for each of the 145,000 molecules in CSD from their size-constrained subsets (all CSD molecules within HAC = query ± 2). A) Recovery of ROCS shape Tanimoto analogs and b) Recovery of ROCS Color Tanimoto analogs.

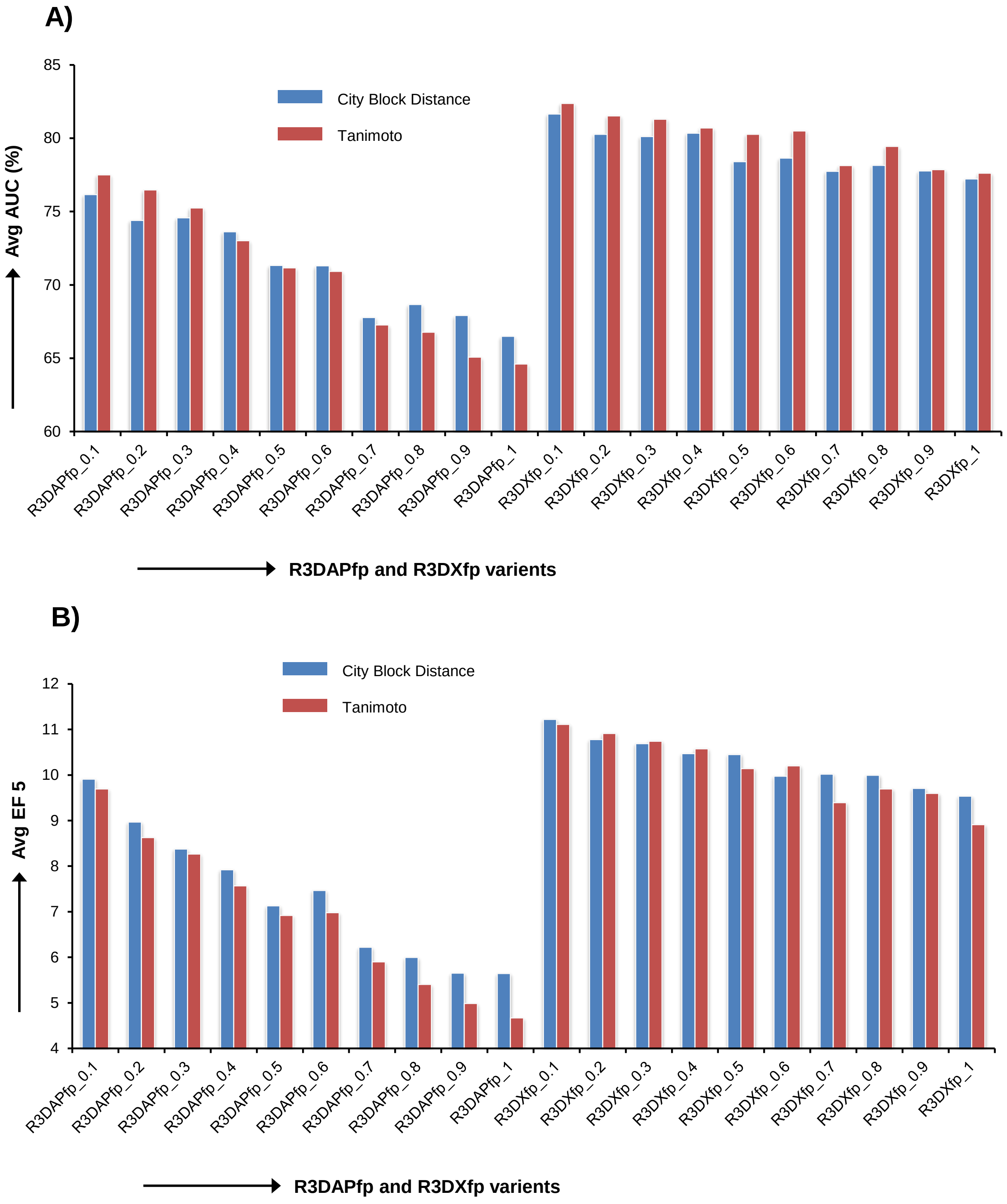

Figure S3. Recovery of 40 set of DUD actives from corresponding decoys set by R3DAPfp and R3DXfp variants using different size of bins during (R3DAPfp_x or R3DXfp_x) construction of fingerprint. City block distance and Tanimoto Coefficient was used as sorting function. Data is represented as average of Area under ROC curve (A) and Enrichment factor at 5% of screen database (B) for 40 targets from DUD.

Table S1. Area under curve (AUC) values for recovery of 40 sets of DUD actives from corresponding decoys by various fingerprints, using City block distance (CBD) as scoring function.

**Target**	3DAPfp	3DXfp	R3DAPfp	R3DXfp	PMIfp	USR	USRCAT	APfp	Xfp
**ace**	38.28	47.96	58.59	49.27	66.86	70.36	62.09	33.13	56.90
**ache**	67.46	81.02	69.99	80.33	62.87	64.89	65.94	60.72	83.90
**ada**	73.88	85.06	78.15	94.89	74.82	62.86	61.14	68.32	90.68
**alr2**	28.67	64.81	30.49	48.18	39.26	50.08	78.38	34.41	30.55
**ampc**	77.22	73.65	86.39	86.16	78.22	79.92	87.91	78.73	85.75
**ar**	73.06	79.08	55.62	81.56	62.84	69.17	39.52	67.35	83.59
**cdk2**	56.49	73.12	57.97	47.52	59.88	67.95	63.47	36.50	41.79
**comt**	53.20	67.36	55.46	72.59	51.87	44.92	41.35	60.72	93.15
**cox1**	41.40	59.47	51.47	60.94	41.26	43.12	49.34	43.04	60.75
**cox2**	85.71	83.15	86.14	91.56	83.61	87.54	90.12	78.28	89.31
**dhfr**	73.36	85.33	78.02	88.03	66.66	67.34	72.30	74.99	89.71
**egfr**	68.76	78.26	73.49	77.70	52.33	65.28	62.93	59.15	77.81
**er_agonist**	85.30	91.10	73.86	80.25	69.77	74.23	90.94	83.39	89.63
**er_antagonist**	72.72	94.12	77.58	81.04	72.60	69.21	82.56	59.14	92.22
**fgfr1**	46.84	79.32	65.81	84.16	37.61	39.93	42.85	38.28	85.71
**fxa**	70.96	92.01	78.19	92.81	66.44	68.34	75.48	69.54	91.74
**gart**	91.27	96.11	93.39	96.15	87.14	87.82	93.93	92.30	97.14
**gpb**	68.46	91.39	79.61	92.03	62.98	58.59	81.98	66.55	90.03
**gr**	67.63	86.77	75.72	75.01	67.34	80.16	68.55	67.48	79.36
**hivpr**	82.10	87.01	81.78	84.80	76.58	79.48	82.81	52.10	70.92
**hivrt**	66.67	72.02	66.31	72.42	73.17	70.20	66.23	57.95	55.41
**hmga**	76.51	88.12	85.29	88.49	74.18	71.31	93.31	76.93	91.78
**hsp90**	89.41	64.62	88.78	68.51	86.93	86.20	90.48	80.65	60.41
**inha**	48.43	86.76	49.44	77.53	57.85	65.45	20.32	38.68	81.88
**mr**	79.94	80.02	79.42	80.43	79.79	85.82	83.60	76.17	78.29
**na**	76.03	92.53	83.59	91.08	80.74	79.37	89.13	73.94	89.73
**p38**	66.67	73.82	75.27	73.34	59.78	66.46	65.02	62.78	70.32
**parp**	46.11	79.74	64.06	80.55	40.27	47.34	55.65	48.44	76.15
**pde5**	68.65	64.78	66.83	62.57	64.41	68.84	55.69	63.63	52.88
**pdgfrb**	47.51	72.59	52.76	63.44	40.03	42.07	28.93	37.55	69.23
**pnp**	77.00	88.48	79.88	86.91	76.55	81.29	80.69	74.08	83.20
**ppar_gamma**	83.52	89.05	86.25	90.74	76.63	80.20	84.72	88.01	91.22
**pr**	65.22	68.34	60.03	62.38	58.23	67.44	76.53	70.24	66.07
**rxr_alpha**	81.15	89.21	89.44	97.35	69.73	81.60	93.17	80.89	95.37
**sahh**	87.26	97.22	88.40	96.44	73.63	69.55	79.93	85.07	97.42
**src**	43.42	65.44	60.38	71.97	41.32	42.45	37.05	43.54	73.23
**thrombin**	73.31	92.81	71.78	89.43	81.37	83.16	89.34	70.88	86.51
**tk**	61.58	85.97	74.37	83.07	41.85	50.90	63.56	60.09	83.72
**trypsin**	73.85	92.01	71.62	89.72	78.45	80.77	86.11	78.12	90.97
**vegfr2**	44.73	51.32	51.92	44.90	41.83	49.31	38.18	42.56	53.04

Table S2. Area under curve (AUC) values for recovery of 40 sets of DUD actives from corresponding decoys by various fingerprints using Tanimoto Coefficient ($T_{fingerprint}$) as scoring function.

Target	3DAPfp	3DXfp	R3DAPfp	R3DXfp	PMIfp	USR	USRCAT	APfp	Xfp
**ace**	36.17	45.42	38.91	50.61	44.50	55.94	42.28	21.71	52.46
**ache**	62.25	73.19	71.40	79.06	61.49	65.77	50.27	60.85	77.64
**ada**	72.90	70.84	80.18	87.30	72.13	63.75	45.72	64.35	76.37
**alr2**	28.60	67.02	40.77	53.30	35.15	49.12	51.73	29.01	47.73
**ampc**	75.19	68.46	81.52	79.52	78.14	79.18	81.99	77.18	74.96
**ar**	72.95	60.26	74.22	86.73	59.38	68.68	33.05	76.57	89.26
**cdk2**	43.91	67.11	46.40	68.76	46.60	63.89	59.42	44.82	58.39
**comt**	50.33	68.33	47.42	81.82	43.60	55.36	32.15	53.31	87.48
**cox1**	40.75	62.18	43.72	61.56	39.89	42.98	46.73	42.65	64.14
**cox2**	85.04	71.64	83.99	84.63	82.89	86.58	64.97	72.28	82.41
**dhfr**	68.44	84.06	79.99	86.13	65.59	66.50	60.28	74.88	86.70
**egfr**	55.37	77.09	63.10	83.36	49.19	62.78	33.30	62.88	78.32
**er_agonist**	85.11	90.81	70.39	81.51	67.16	71.94	75.25	70.88	90.26
**er_antagonist**	61.58	92.06	69.11	94.25	69.74	68.33	71.99	49.77	92.26
**fgfr1**	41.07	67.79	75.20	87.57	33.17	37.44	51.44	38.32	83.02
**fxa**	70.08	88.28	77.09	92.14	64.91	66.41	68.72	68.33	89.31
**gart**	89.73	95.85	93.44	95.45	85.78	86.79	93.58	92.07	96.68
**gpb**	64.94	91.08	77.92	92.25	58.00	55.65	80.03	52.66	89.52
**gr**	63.57	86.66	72.48	71.52	66.87	75.69	49.13	54.07	81.36
**hivpr**	65.77	73.92	80.33	82.81	74.92	76.25	76.83	53.72	62.59
**hivrt**	57.46	58.35	65.19	77.06	70.43	69.39	59.21	44.56	59.85
**hmga**	77.05	79.85	83.77	92.76	69.32	69.36	90.98	77.80	93.76
**hsp90**	88.39	64.87	89.77	69.23	87.90	84.20	89.91	77.60	67.17
**inha**	47.84	76.51	49.60	78.78	58.89	67.43	32.52	37.09	70.01
**mr**	79.58	76.22	84.79	84.10	78.38	82.68	54.71	75.30	86.82
**na**	75.14	91.75	82.84	90.84	75.75	74.44	86.75	60.83	85.40
**p38**	66.75	75.12	75.50	73.85	57.86	67.77	49.59	59.45	69.14
**parp**	52.14	79.37	65.56	84.69	38.06	47.05	57.74	47.61	78.65
**pde5**	67.93	47.91	68.65	58.39	60.78	72.73	44.08	59.63	53.55
**pdgfrb**	41.49	79.25	58.65	75.44	35.71	42.08	32.12	32.39	70.43
**pnp**	77.64	82.52	81.83	88.88	75.94	79.76	74.65	72.57	79.24
**ppar_gamma**	80.53	85.32	86.07	87.85	77.10	77.99	82.80	86.72	88.67
**pr**	64.63	87.89	57.60	63.94	56.32	65.35	70.53	65.23	77.65
**rxr_alpha**	79.69	85.97	90.59	95.61	71.29	80.05	84.93	74.89	90.90
**sahh**	87.22	94.55	93.25	93.93	73.54	71.11	69.05	86.53	97.30
**src**	39.86	64.81	73.14	76.30	32.25	39.88	37.41	42.25	72.13
**thrombin**	71.44	87.94	68.89	85.99	79.24	79.58	80.28	64.06	83.72
**tk**	56.62	85.23	80.38	88.13	42.93	45.41	58.82	54.88	82.25
**trypsin**	74.54	88.55	71.57	89.47	74.12	75.71	77.99	69.46	90.02
**vegfr2**	46.24	55.77	51.50	55.38	40.30	48.65	34.10	44.89	63.39

Table S3. Enrichment factors at 5 % of screen database for recovery of 40 sets of DUD actives from corresponding decoys by various fingerprints using City block distance ($CBD_{fingerprint}$) as scoring function.

Target	3DAPfp	3DXfp	R3DAPfp	R3DXfp	PMIfp	USR	USRCAT	APfp	Xfp
**ace**	1.65	3.71	6.18	3.29	2.06	3.29	5.35	1.65	6.18
**ache**	6.32	7.47	7.85	9.19	4.79	4.40	8.04	3.45	9.95
**ada**	7.86	12.23	9.61	13.11	5.24	5.24	6.12	7.86	11.36
**alr2**	0.77	3.09	0.77	3.09	0.77	2.32	4.64	0.77	3.09
**ampc**	6.78	10.66	10.66	11.63	4.85	4.85	13.57	9.69	10.66
**ar**	5.68	2.98	6.22	7.30	2.70	5.95	5.41	4.60	7.30
**cdk2**	6.03	3.22	4.02	0.80	2.01	2.01	1.61	0.80	0.40
**comt**	5.47	9.11	5.47	9.11	1.82	1.82	1.82	5.47	14.58
**cox1**	0.81	4.88	4.88	6.50	2.44	3.25	2.44	1.63	4.88
**cox2**	6.90	10.24	7.25	11.22	5.64	10.47	11.27	8.40	10.01
**dhfr**	5.78	10.55	6.47	12.55	1.59	1.19	6.07	7.17	11.45
**egfr**	5.77	7.44	8.61	9.02	1.62	3.34	3.56	1.98	7.44
**er_agonist**	9.86	15.83	9.26	10.76	4.18	5.68	14.34	10.46	16.13
**er_antagonist**	1.04	12.43	3.11	7.77	5.18	2.07	10.36	3.11	12.43
**fgfr1**	3.05	4.41	5.43	11.53	1.53	1.36	3.05	3.22	8.82
**fxa**	5.09	13.85	7.91	14.13	2.26	2.26	7.49	6.78	15.12
**gart**	9.70	16.49	16.49	16.49	7.76	6.79	17.46	8.73	16.49
**gpb**	6.06	15.76	10.51	16.16	1.62	3.23	13.72	4.85	13.74
**gr**	2.06	9.28	9.28	11.86	1.03	3.35	6.19	6.70	12.63
**hivpr**	4.57	9.14	4.57	10.67	3.43	3.05	7.62	6.86	12.57
**hivrt**	4.55	3.54	5.06	7.59	5.56	3.03	2.53	5.06	3.03
**hmga**	4.05	10.42	11.57	13.89	1.16	1.16	17.94	8.10	16.78
**hsp90**	8.37	10.88	10.88	10.88	7.53	5.86	8.37	5.02	10.88
**inha**	1.54	8.24	2.06	7.98	1.29	2.57	0.77	2.57	9.01
**mr**	10.86	10.86	12.22	9.51	6.79	12.22	9.51	9.51	10.86
**na**	4.52	15.21	10.69	13.98	4.93	5.76	8.63	7.40	13.98
**p38**	4.45	4.69	8.60	7.35	1.64	2.89	5.55	3.36	7.50
**parp**	2.44	10.38	4.88	9.77	1.83	1.22	6.11	2.44	8.55
**pde5**	0.78	2.35	0.78	5.49	1.57	1.57	1.18	1.96	1.18
**pdgfrb**	2.94	6.76	3.19	8.17	1.91	2.68	2.17	2.42	5.87
**pnp**	4.84	15.32	8.06	15.32	5.64	6.45	12.90	2.42	12.90
**ppar_gamma**	11.63	16.33	13.36	17.08	7.92	9.90	14.60	14.60	17.82
**pr**	4.51	3.76	4.51	11.27	3.01	4.51	14.28	8.26	11.27
**rxr_alpha**	10.11	12.13	16.18	17.19	1.01	10.11	12.13	10.11	13.14
**sahh**	8.57	16.53	6.73	16.53	1.22	4.29	7.33	4.29	15.92
**src**	2.97	5.30	4.26	9.69	1.81	2.20	3.49	3.10	8.66
**thrombin**	2.52	14.47	2.83	12.27	4.40	5.98	12.09	5.98	14.16
**tk**	3.66	7.33	7.33	10.08	0.92	0.92	4.58	5.50	6.41
**trypsin**	2.74	14.62	3.65	14.16	3.65	6.40	11.42	9.59	16.45
**vegfr2**	0.82	1.63	3.26	3.53	1.36	2.17	1.63	2.45	2.99

Table S4. Enrichment factors at 5 % of screen database for recovery of 40 sets of DUD actives from corresponding decoys by various fingerprints using Tanimoto Coefficient ($T_{fingerprint}$) as scoring function.

Target	3DAPfp	3DXfp	R3DAPfp	R3DXfp	PMIfp	USR	USRCAT	APfp	Xfp
**ace**	1.24	2.47	1.65	3.71	1.65	0.41	2.06	0.82	5.77
**ache**	3.25	8.81	9.76	7.66	3.06	2.87	1.72	3.06	8.61
**ada**	8.74	11.36	10.48	12.23	2.62	5.24	2.62	6.99	11.36
**alr2**	0.77	2.32	1.55	3.09	1.55	1.55	6.95	0.77	3.09
**ampc**	5.81	9.69	11.63	10.66	4.85	3.88	8.72	8.72	9.69
**ar**	5.68	6.22	5.68	9.20	2.16	6.22	4.06	3.52	7.30
**cdk2**	0.80	3.62	0.80	4.82	0.40	2.01	2.01	2.01	3.22
**comt**	3.64	7.29	5.47	9.11	1.82	5.47	1.82	5.47	10.93
**cox1**	1.63	4.07	1.63	6.50	2.44	3.25	3.25	1.63	7.32
**cox2**	7.31	9.55	7.08	9.43	5.81	8.34	8.92	5.18	9.43
**dhfr**	3.29	8.07	6.37	8.96	1.39	1.29	2.79	8.36	9.76
**egfr**	2.39	6.63	2.62	9.29	1.62	1.62	0.59	2.89	5.86
**er_agonist**	8.96	14.94	8.96	9.56	1.49	3.59	12.25	2.09	14.94
**er_antagonist**	1.55	11.39	4.14	13.46	1.55	1.55	10.88	1.55	12.43
**fgfr1**	2.37	3.05	7.46	9.84	0.68	1.19	3.22	3.73	7.97
**fxa**	5.09	13.28	8.05	13.99	2.97	2.12	6.64	5.23	13.57
**gart**	7.76	15.52	16.49	15.52	7.76	5.82	17.46	9.70	16.49
**gpb**	6.06	14.55	9.70	16.16	1.62	2.83	8.47	3.23	11.31
**gr**	1.55	8.25	7.99	11.08	0.77	2.58	0.77	2.58	9.79
**hivpr**	0.76	11.43	5.33	7.62	3.81	3.05	3.43	7.62	7.62
**hivrt**	1.01	2.53	5.06	7.08	3.54	3.54	1.52	2.02	4.05
**hmga**	6.37	5.79	12.15	15.62	2.89	2.31	15.05	8.10	16.78
**hsp90**	7.53	10.88	13.39	10.88	8.37	1.67	7.53	4.19	10.88
**inha**	1.29	9.52	2.06	10.55	1.54	2.32	1.03	0.77	9.52
**mr**	10.86	6.79	12.22	8.15	5.43	5.43	6.79	5.43	8.15
**na**	4.52	15.21	10.69	15.21	2.88	1.23	7.81	3.29	13.57
**p38**	4.06	5.31	8.99	7.50	1.25	2.74	3.83	3.52	6.95
**parp**	3.05	8.55	5.50	10.38	1.83	1.22	5.50	2.44	8.55
**pde5**	1.18	1.96	1.57	2.75	1.57	0.78	0.39	1.57	2.35
**pdgfrb**	2.04	7.02	3.06	5.36	1.02	2.42	1.28	1.66	7.02
**pnp**	5.64	13.71	6.45	15.32	5.64	6.45	12.09	3.22	14.51
**ppar_gamma**	9.40	15.34	12.87	17.08	7.92	8.41	14.35	12.87	17.08
**pr**	3.76	12.77	5.26	11.27	3.76	3.76	13.52	5.26	12.77
**rxr_alpha**	9.10	10.11	15.17	13.14	3.03	9.10	9.10	5.06	12.13
**sahh**	7.96	13.47	7.35	14.69	1.22	3.06	7.95	4.29	17.75
**src**	1.68	4.13	5.56	9.95	0.13	2.07	3.23	2.97	6.59
**thrombin**	2.52	11.96	3.15	12.90	3.78	5.03	9.61	3.46	14.16
**tk**	3.66	6.41	7.33	8.24	0.92	0.92	4.58	0.92	6.41
**trypsin**	3.20	13.71	3.65	14.62	1.83	4.57	9.14	4.11	15.53
**vegfr2**	1.36	2.99	2.45	2.99	1.63	2.45	1.63	2.99	2.45

Table S5. Area under curve (AUC) values for recovery of 40 sets of DUD actives from ZINC by various fingerprints using City block distance (CBD) as scoring function.

**Target**	3DAPfp	3DXfp	R3DAPfp	R3DXfp	PMIfp	USR	USRCAT	APfp	Xfp
**ace**	53.98	45.81	65.67	57.81	68.80	69.35	69.25	48.66	65.85
**ache**	72.88	85.79	75.57	88.05	66.26	68.47	74.15	69.33	88.95
**ada**	92.97	94.91	95.73	98.10	92.91	85.91	93.53	91.66	98.83
**alr2**	65.12	78.05	73.85	73.67	59.46	72.88	85.65	75.51	53.24
**ampc**	92.22	71.28	94.67	93.51	90.18	91.23	95.42	93.17	93.36
**ar**	89.42	83.99	80.13	89.15	83.09	85.42	67.95	89.97	90.86
**cdk2**	63.87	80.66	62.77	51.85	63.94	69.14	85.54	53.30	49.07
**comt**	73.56	82.64	78.72	91.58	69.68	67.64	71.34	76.88	99.50
**cox1**	75.06	85.02	82.38	90.66	71.35	71.14	87.11	74.72	90.00
**cox2**	93.94	84.29	95.22	96.36	89.27	89.50	91.61	92.17	96.16
**dhfr**	80.89	91.03	87.21	84.82	69.81	67.98	86.63	83.56	84.66
**egfr**	77.04	77.27	86.15	82.57	58.52	66.48	75.19	73.71	82.09
**er_agonist**	96.64	92.92	91.85	87.84	90.73	90.25	98.43	96.96	93.29
**er_antagonist**	90.28	93.83	93.18	83.22	86.60	76.16	94.25	87.95	97.99
**fgfr1**	55.44	78.92	73.54	81.19	39.69	37.78	56.79	61.36	90.00
**fxa**	82.44	91.33	89.41	89.88	68.04	65.18	88.72	88.82	94.58
**gart**	96.82	98.62	96.19	96.13	96.38	95.81	99.37	99.13	99.92
**gpb**	89.92	95.88	91.44	96.13	87.97	86.00	95.99	88.11	95.36
**gr**	94.46	94.24	95.22	92.47	87.89	91.88	88.78	95.56	93.26
**hivpr**	83.72	65.01	92.69	78.30	69.39	69.59	83.59	91.23	75.38
**hivrt**	79.30	71.07	80.51	75.37	78.75	79.12	83.36	75.43	63.01
**hmga**	83.90	75.31	89.63	79.15	71.04	66.94	95.29	86.68	88.24
**hsp90**	97.06	79.34	95.96	75.74	95.19	88.91	99.14	96.14	73.27
**inha**	62.88	87.82	65.05	81.53	66.35	70.22	51.43	53.26	87.22
**mr**	93.57	78.49	94.26	82.40	91.03	93.55	93.53	88.62	80.46
**na**	86.38	96.03	90.16	92.84	88.54	85.84	95.75	85.70	92.20
**p38**	80.59	80.14	89.14	91.33	68.71	71.09	80.29	83.18	90.29
**parp**	87.89	89.87	94.64	96.50	82.02	81.04	84.38	90.81	94.07
**pde5**	77.90	46.05	78.52	42.74	61.07	57.25	64.59	78.55	66.73
**pdgfrb**	57.67	62.79	68.77	61.60	47.75	46.44	54.13	50.27	76.56
**pnp**	90.34	92.58	92.70	89.79	89.27	91.92	93.91	87.94	86.18
**ppar_gamma**	96.99	94.61	97.13	96.92	92.03	87.96	92.64	97.16	95.60
**pr**	81.53	84.93	73.61	84.97	74.86	80.54	88.24	79.04	87.77
**rxr_alpha**	94.10	93.48	97.95	99.73	83.78	85.40	98.46	94.94	99.78
**sahh**	98.29	100.00	98.49	99.49	92.55	88.27	99.44	98.32	99.99
**src**	51.96	68.52	72.30	73.60	38.49	39.15	55.41	62.97	78.51
**thrombin**	73.30	94.78	81.22	87.91	74.69	77.69	92.34	84.78	92.85
**tk**	96.35	99.97	97.10	95.07	82.25	89.01	93.81	92.99	99.26
**trypsin**	84.74	91.99	83.92	87.13	74.53	75.39	90.61	92.31	94.17
**vegfr2**	57.90	59.34	64.22	57.37	51.26	50.60	63.40	63.17	66.37

Table S6. Area under curve (AUC) values for recovery of 40 sets of DUD actives from ZINC by various fingerprints using Tanimoto Coefficient ($T_{fingerprint}$) as scoring function.

Target	3DAPfp	3DXfp	R3DAPfp	R3DXfp	PMIfp	USR	USRCAT	APfp	Xfp
**ace**	50.95	72.16	55.13	56.39	56.95	59.95	46.30	36.50	60.22
**ache**	69.33	80.06	76.89	88.63	65.33	68.72	63.86	71.05	84.90
**ada**	91.27	85.75	92.72	98.10	90.09	84.38	89.04	87.55	92.55
**alr2**	61.36	67.63	64.05	73.52	54.59	70.58	60.67	72.42	59.24
**ampc**	91.00	65.24	90.37	91.33	90.28	90.17	92.41	91.50	78.47
**ar**	91.30	65.33	91.46	93.85	81.14	83.98	48.21	93.16	97.39
**cdk2**	59.67	90.95	64.67	82.56	56.05	64.77	85.38	60.26	78.61
**comt**	70.80	93.58	73.69	98.75	66.08	69.06	62.50	77.20	97.16
**cox1**	73.92	81.51	66.42	89.88	67.35	66.03	80.43	74.03	83.39
**cox2**	93.74	69.87	94.76	92.45	89.01	88.60	72.48	89.87	91.83
**dhfr**	76.98	96.62	88.68	93.87	69.40	66.00	82.22	83.45	95.30
**egfr**	70.41	84.69	83.77	92.06	58.32	64.10	66.67	76.97	88.20
**er_agonist**	96.84	93.00	85.83	90.66	89.92	87.88	81.81	94.51	96.40
**er_antagonist**	90.07	95.68	95.17	99.50	91.23	74.05	87.49	91.32	98.67
**fgfr1**	53.77	90.40	85.57	93.73	46.90	42.57	80.13	65.53	92.42
**fxa**	83.05	94.63	91.24	95.81	70.32	64.04	92.45	90.21	96.71
**gart**	97.41	99.91	97.40	99.91	96.14	94.90	99.15	99.00	99.97
**gpb**	89.22	97.41	90.20	96.82	85.78	83.66	95.42	82.67	96.60
**gr**	94.10	94.60	93.84	91.55	87.83	90.40	80.13	93.18	95.17
**hivpr**	90.44	68.22	95.80	89.99	70.76	67.72	88.26	92.62	81.87
**hivrt**	75.04	72.91	78.61	80.65	78.60	77.71	82.63	65.97	72.74
**hmga**	87.22	90.36	90.41	93.01	70.81	63.55	95.66	87.75	93.17
**hsp90**	96.98	85.29	96.70	84.22	95.53	90.86	99.17	94.04	87.75
**inha**	63.15	82.43	68.10	90.18	68.41	71.19	60.12	56.20	86.19
**mr**	94.01	68.71	97.70	92.22	90.71	90.70	87.34	95.20	96.17
**na**	86.34	97.15	91.05	95.43	86.48	82.78	95.93	78.74	91.28
**p38**	81.40	84.68	89.91	91.57	67.47	71.39	73.83	82.39	92.66
**parp**	89.04	89.02	95.44	96.62	77.56	77.89	77.73	88.52	92.86
**pde5**	78.56	73.08	85.36	74.91	60.84	61.20	64.51	81.13	78.18
**pdgfrb**	54.53	75.06	78.03	89.06	49.19	49.28	55.20	52.74	87.51
**pnp**	88.66	96.53	90.75	93.86	86.26	89.15	86.65	85.25	84.54
**ppar_gamma**	96.95	90.80	97.06	95.63	92.82	86.46	91.09	97.29	93.93
**pr**	84.16	90.52	71.20	86.81	73.45	76.66	78.72	80.93	95.39
**rxr_alpha**	94.20	83.24	99.04	99.48	87.62	85.09	91.82	96.66	97.25
**sahh**	98.13	99.97	99.83	99.23	92.50	87.27	94.81	98.82	99.98
**src**	56.04	78.53	84.68	83.64	41.22	40.48	64.74	66.45	83.71
**thrombin**	74.14	96.48	85.16	93.60	76.18	74.85	90.59	85.51	93.90
**tk**	94.57	99.93	99.08	98.69	81.93	87.49	91.51	93.09	99.25
**trypsin**	90.36	95.66	89.36	94.68	80.91	71.30	87.25	89.92	96.24
**vegfr2**	59.00	75.11	70.31	77.91	53.42	51.58	66.79	65.90	81.66

Table S7. Enrichment factors at 0.1 % of screen database for recovery of 40 sets of DUD actives from ZINC by various fingerprints using City block distance ($CBD_{fingerprint}$) as scoring function.

Target	3DAPfp	3DXfp	R3DAPfp	R3DXfp	PMIfp	USR	USRCAT	APfp	Xfp
**ace**	40.82	122.45	40.82	102.04	20.41	40.82	81.63	20.41	224.49
**ache**	142.86	228.57	228.57	352.39	19.05	66.67	285.72	133.34	314.29
**ada**	347.83	695.66	478.27	304.35	43.48	86.96	434.79	260.87	565.22
**alr2**	38.46	153.85	38.46	153.85	38.46	38.46	38.46	38.46	153.85
**ampc**	238.10	476.19	333.34	523.81	95.24	190.48	666.67	95.24	476.19
**ar**	162.16	54.05	256.76	27.03	40.54	148.65	216.22	189.19	40.54
**cdk2**	20.00	80.00	20.00	20.00	20.00	20.00	40.00	20.00	20.00
**comt**	181.82	454.55	272.73	363.64	90.91	90.91	181.82	90.91	727.28
**cox1**	40.00	240.00	40.00	120.00	80.00	120.00	120.00	40.00	200.00
**cox2**	192.53	416.68	126.44	359.20	8.62	201.15	270.12	270.12	359.20
**dhfr**	74.63	457.72	124.38	263.69	4.98	4.98	248.76	99.50	134.33
**egfr**	137.39	128.38	173.43	231.99	2.25	27.03	69.82	13.51	112.62
**er_agonist**	328.36	358.21	373.14	149.26	44.78	104.48	611.95	462.69	582.10
**er_antagonist**	51.28	512.83	51.28	205.13	25.64	25.64	461.54	51.28	564.11
**fgfr1**	84.75	110.17	169.49	296.61	25.42	42.37	93.22	84.75	161.02
**fxa**	161.97	507.05	345.08	514.09	42.25	63.38	309.86	183.10	563.39
**gart**	619.05	952.39	809.53	952.39	333.34	476.19	952.39	380.96	904.77
**gpb**	220.00	860.01	360.00	840.01	80.00	120.00	700.01	120.00	920.01
**gr**	76.92	435.90	371.80	589.75	12.82	89.74	307.70	346.16	641.03
**hivpr**	18.87	150.95	169.81	169.81	18.87	18.87	169.81	358.49	301.89
**hivrt**	50.00	100.00	175.00	75.00	25.00	50.00	25.00	200.00	50.00
**hmga**	142.86	314.29	400.00	457.15	28.57	28.57	285.72	314.29	542.86
**hsp90**	250.00	541.67	458.34	541.67	166.67	125.00	416.67	125.00	541.67
**inha**	38.46	282.05	38.46	192.31	25.64	25.64	12.82	64.10	282.05
**mr**	800.01	333.34	666.67	466.67	133.33	200.00	466.67	666.67	533.34
**na**	102.04	714.29	204.08	653.07	20.41	81.63	306.13	142.86	653.07
**p38**	89.85	144.53	359.38	289.07	19.53	11.72	179.69	128.91	339.85
**parp**	121.21	181.82	272.73	454.55	30.30	60.61	242.43	121.21	424.25
**pde5**	19.61	19.61	19.61	39.22	39.22	19.61	19.61	19.61	39.22
**pdgfrb**	38.22	44.59	50.96	114.65	12.74	50.96	89.17	38.22	12.74
**pnp**	120.00	600.01	360.00	560.01	120.00	240.00	640.01	40.00	400.00
**ppar_gamma**	691.37	814.82	765.44	864.21	395.07	345.68	740.75	876.55	888.90
**pr**	74.07	185.19	37.04	555.56	37.04	74.07	666.67	407.41	555.56
**rxr_alpha**	300.00	600.01	750.01	850.01	50.00	200.00	600.00	400.00	650.01
**sahh**	636.37	969.71	393.94	848.49	30.30	121.21	606.07	272.73	939.40
**src**	96.78	109.68	167.74	290.33	6.45	64.52	109.68	122.58	154.84
**thrombin**	15.63	515.63	31.25	453.13	31.25	78.13	538.47	109.38	656.26
**tk**	181.82	863.64	363.64	590.91	45.45	45.45	454.55	318.18	727.28
**trypsin**	45.46	568.19	45.46	500.01	22.73	136.37	568.19	250.00	704.55
**vegfr2**	27.03	13.51	94.60	27.03	27.03	13.51	40.54	40.54	13.51

Table S8. Enrichment factors at 0.1 % of screen database for recovery of 40 sets of DUD actives from ZINC by various fingerprints using Tanimoto Coefficient ($T_{fingerprint}$) as scoring function.

Target	3DAPfp	3DXfp	R3DAPfp	R3DXfp	PMIfp	USR	USRCAT	APfp	Xfp
**ace**	40.82	81.63	40.82	102.04	20.41	20.41	20.41	40.82	285.72
**ache**	28.57	323.81	200.00	238.10	19.05	9.52	19.05	104.76	257.15
**ada**	347.83	565.22	434.79	608.70	43.48	86.96	217.39	130.44	608.70
**alr2**	38.46	76.92	38.46	153.85	38.46	38.46	115.39	38.46	153.85
**ampc**	190.48	380.96	238.10	523.81	95.24	190.48	428.57	95.24	333.34
**ar**	256.76	216.22	256.76	13.51	27.03	121.62	175.68	364.87	364.87
**cdk2**	20.00	140.00	20.00	120.00	20.00	20.00	40.00	20.00	140.00
**comt**	90.91	363.64	363.64	454.55	90.91	181.82	181.82	90.91	636.37
**cox1**	40.00	200.00	80.00	80.00	80.00	40.00	120.00	40.00	40.00
**cox2**	163.80	258.63	163.80	370.70	8.62	152.30	227.02	178.17	209.77
**dhfr**	59.70	363.19	129.36	318.41	4.98	4.98	94.53	54.73	353.24
**egfr**	29.28	78.83	56.31	168.92	6.76	4.50	11.26	74.33	42.79
**er_agonist**	328.36	552.24	388.06	29.85	14.93	104.48	552.24	44.78	626.87
**er_antagonist**	51.28	487.18	76.92	666.67	76.92	25.64	307.70	25.64	538.47
**fgfr1**	67.80	110.17	194.92	296.61	16.95	42.37	101.70	84.75	67.80
**fxa**	112.68	605.64	345.08	514.09	21.13	63.38	316.91	161.97	584.52
**gart**	619.05	952.39	809.53	952.39	47.62	142.86	952.39	380.96	952.39
**gpb**	120.00	900.01	360.00	940.01	80.00	20.00	260.00	60.00	940.01
**gr**	76.92	410.26	320.52	564.11	12.82	38.46	38.46	128.21	589.75
**hivpr**	37.74	113.21	207.55	150.95	18.87	18.87	75.47	377.36	301.89
**hivrt**	25.00	75.00	175.00	75.00	25.00	25.00	25.00	50.00	150.00
**hmga**	142.86	228.57	457.15	457.15	57.14	28.57	257.15	314.29	400.00
**hsp90**	250.00	541.67	500.00	541.67	41.67	41.67	416.67	208.34	541.67
**inha**	25.64	230.77	38.46	461.54	25.64	25.64	12.82	25.64	397.44
**mr**	800.01	333.34	800.01	400.00	66.67	133.33	333.34	800.01	400.00
**na**	81.63	632.66	204.08	673.48	20.41	20.41	244.90	40.82	551.03
**p38**	74.22	3.91	394.54	273.44	15.63	11.72	132.82	46.88	242.19
**parp**	90.91	181.82	272.73	393.94	60.61	60.61	90.91	121.21	424.25
**pde5**	19.61	39.22	19.61	19.61	39.22	19.61	19.61	19.61	19.61
**pdgfrb**	44.59	38.22	50.96	159.24	6.37	44.59	25.48	50.96	121.02
**pnp**	120.00	680.01	320.00	640.01	120.00	200.00	600.01	80.00	600.01
**ppar_gamma**	666.67	716.06	790.13	851.86	395.07	234.57	691.37	802.48	839.52
**pr**	74.07	481.49	74.07	185.19	37.04	148.15	555.56	148.15	555.56
**rxr_alpha**	200.00	500.00	750.01	500.00	50.00	100.00	450.00	250.00	500.00
**sahh**	606.07	909.10	515.16	787.89	30.30	121.21	515.16	303.03	909.10
**src**	6.45	109.68	174.20	277.42	6.45	19.36	51.61	129.03	148.39
**thrombin**	15.63	546.88	31.25	515.63	15.63	93.75	430.77	31.25	671.88
**tk**	181.82	772.73	409.09	590.91	45.45	45.45	363.64	45.45	772.73
**trypsin**	22.73	681.83	45.46	590.92	22.73	22.73	431.82	22.73	772.74
**vegfr2**	27.03	40.54	94.60	40.54	13.51	13.51	40.54	40.54	54.05

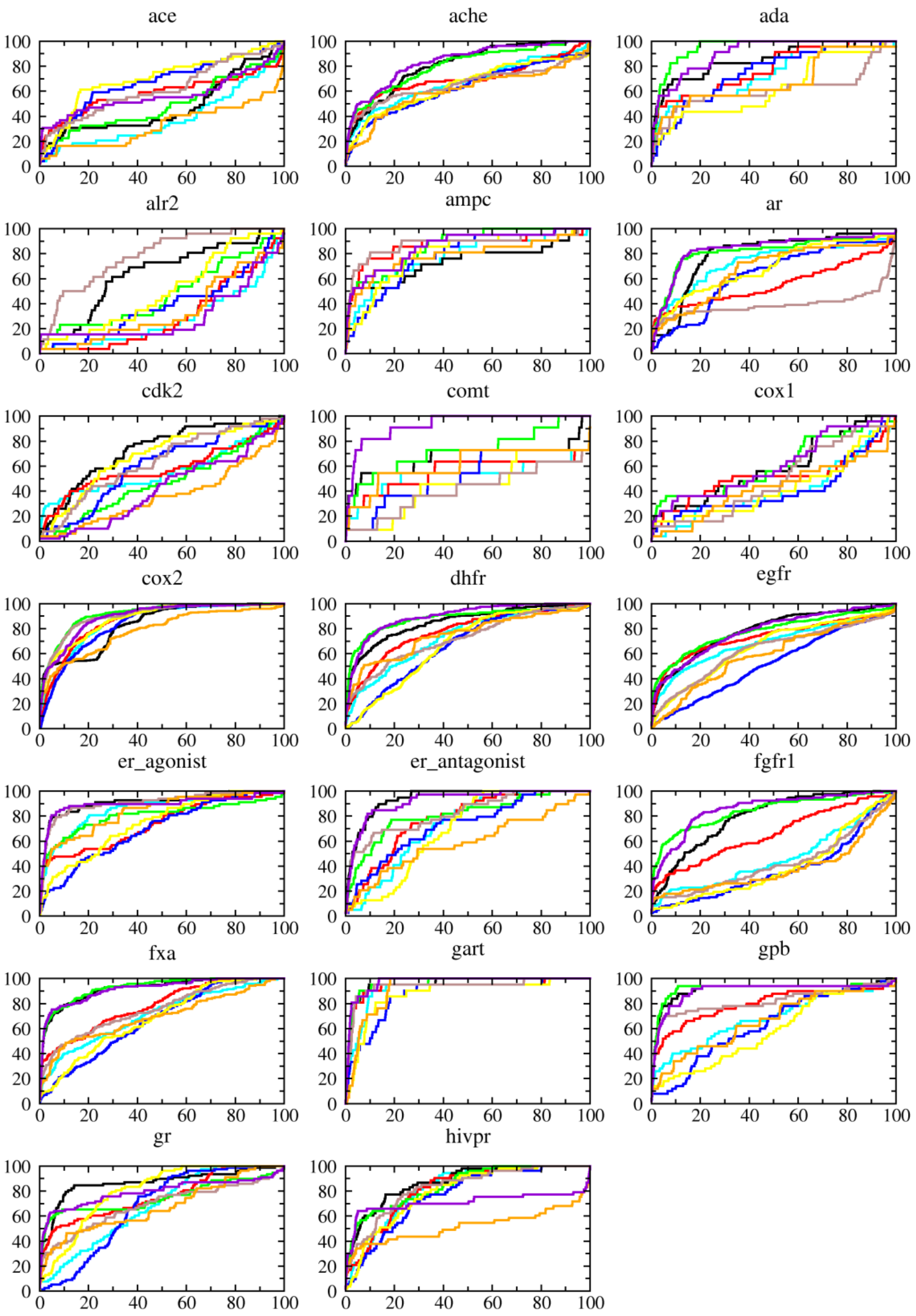
ace
ache
ada
alr2
ampc
ar
cdk2
comt
cox1
cox2
dhfr
egfr
er_agonist
er_antagonist
fgfr1
fxa
gart
gpb
gr
hivpr
0
20
40
60
80
100

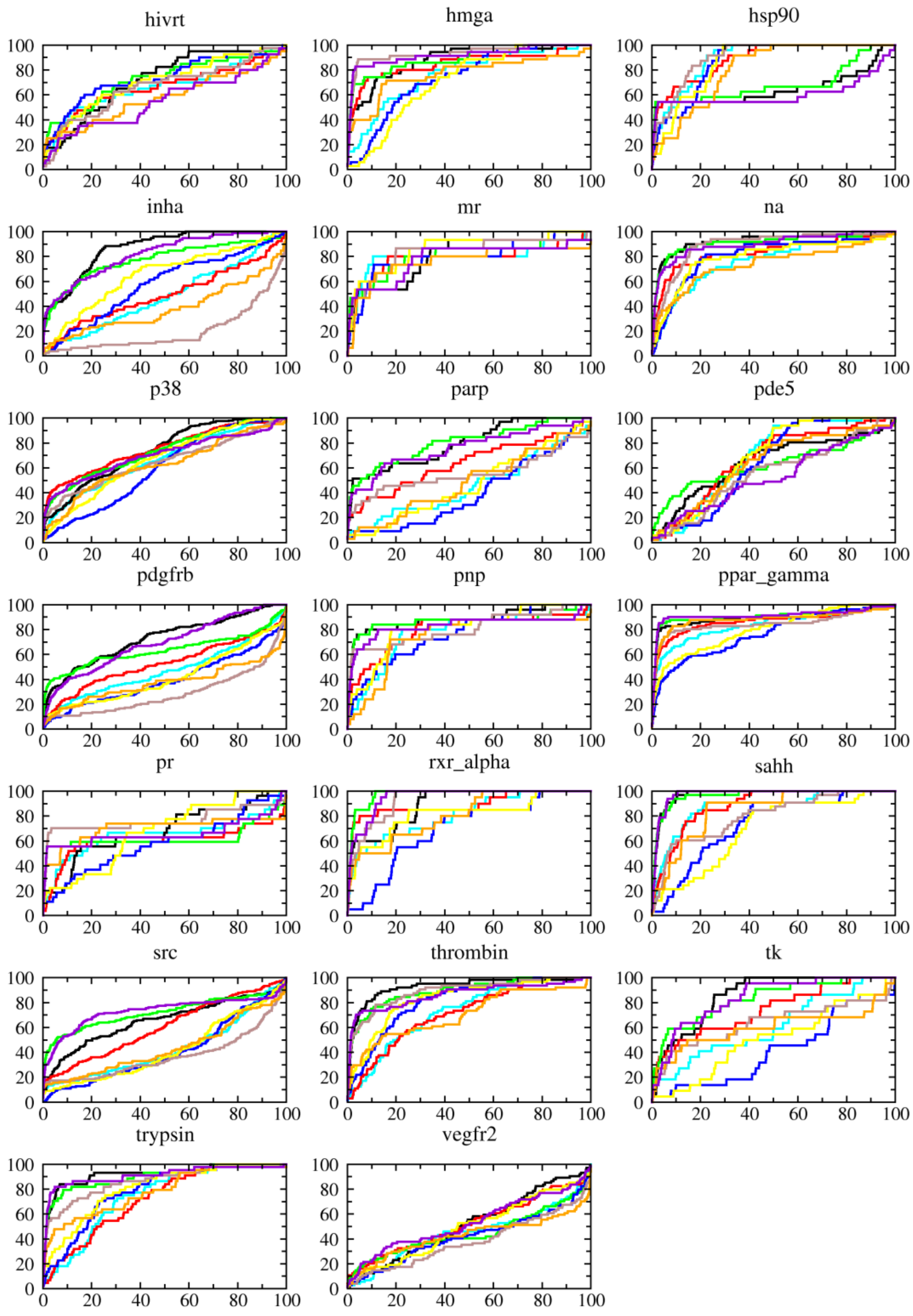

Figure S4. Receiver Operating Characteristic (ROC) curves for recovery of DUD actives from decoys using various fingerprints. City block distance ($CBD_{fingerprint}$) was used as scoring function. 3DAPfp=Cyan; 3DXfp=Black; R3DAPfp=Red; R3DXfp=Green; PMIfp=Blue; USR=Yellow; USRCAT=Brown; APfp=Orange; Xfp=Violet. X-axis is % of sorted database and Y-axis=% of actives found.

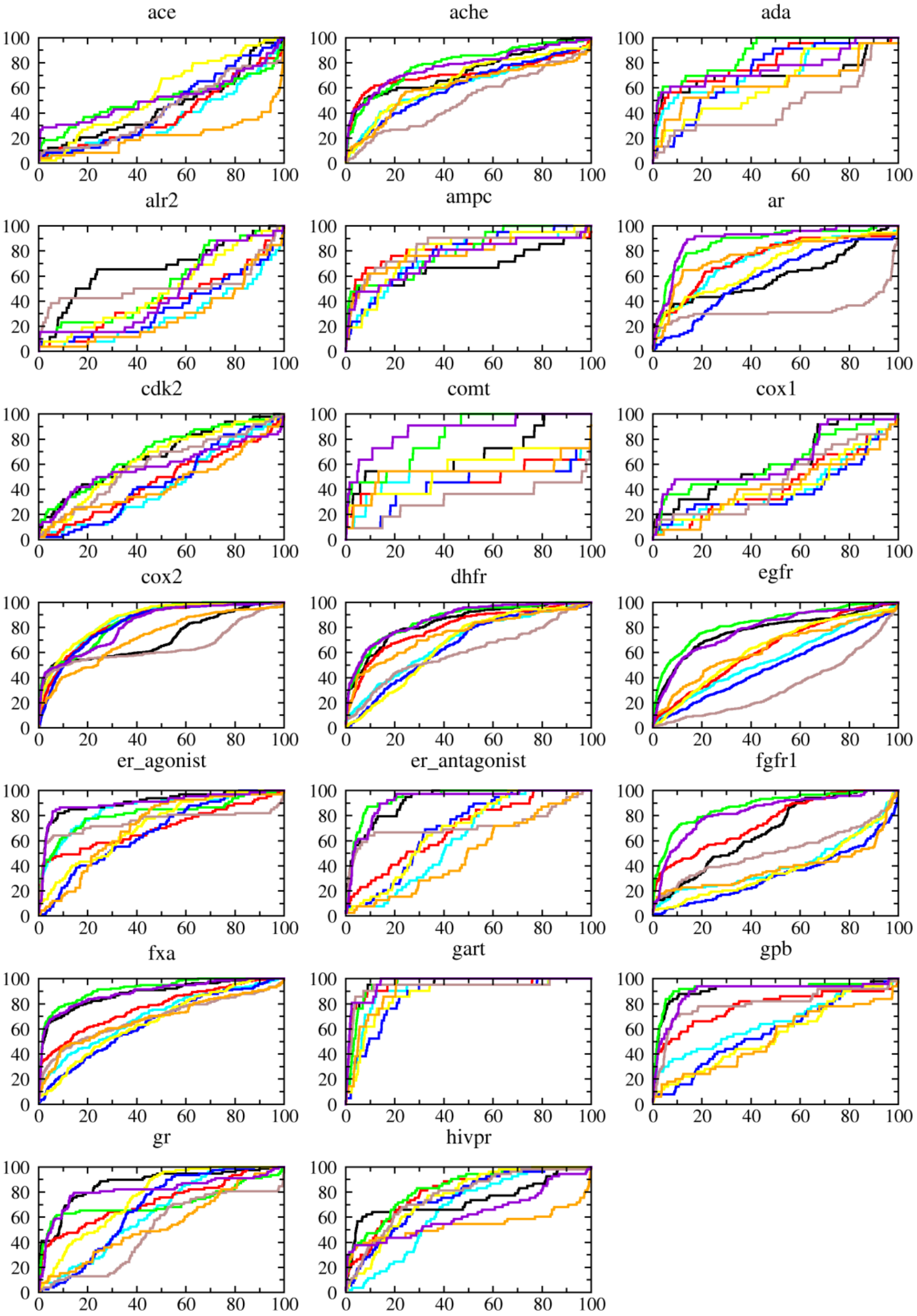
ace
ache
ada
alr2
ampc
ar
cdk2
comt
cox1
cox2
dhfr
egfr
er_agonist
er_antagonist
fgfr1
fxa
gart
gpb
gr
hivpr

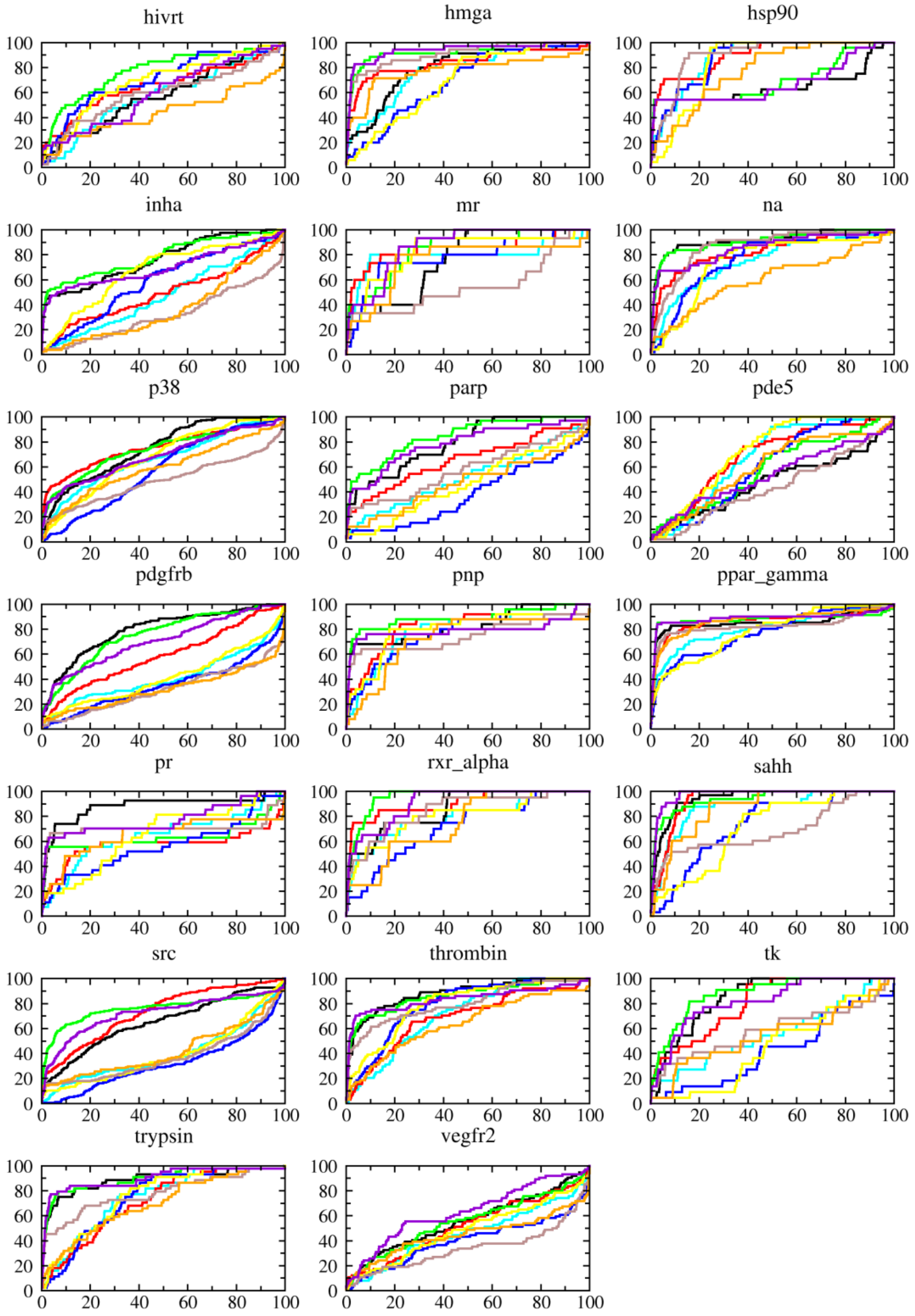

Figure S5. Receiver Operating Characteristic (ROC) curves for recovery of DUD actives from decoys using various fingerprints. Tanimoto coefficient ($T_{\mathrm{fingerprint}}$) was used as scoring function. 3DAPfp=Cyan; 3DXfp=Black; R3DAPfp=Red; R3DXfp=Green; PMIfp=Blue; USR=Yellow; USRCAT=Brown; APfp=Orange; Xfp=Violet. X-axis is % of sorted database and Y-axis=% of actives found.

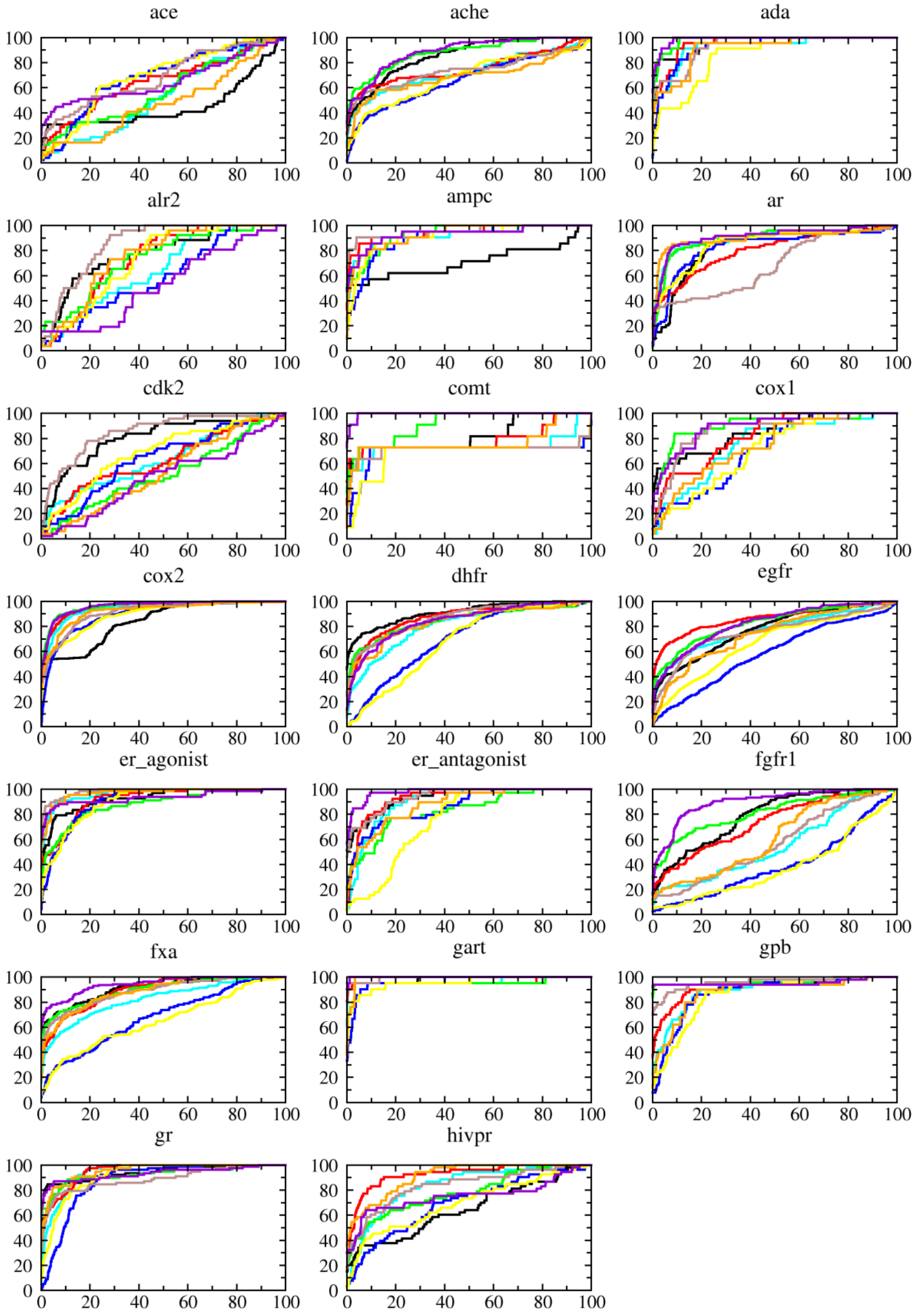
ace
ache
ada
alr2
ampc
ar
cdk2
comt
cox1
cox2
dhfr
egfr
er_agonist
er_antagonist
fgfr1
fxa
gart
gpb
gr
hivpr
0 20 40 60 80 100

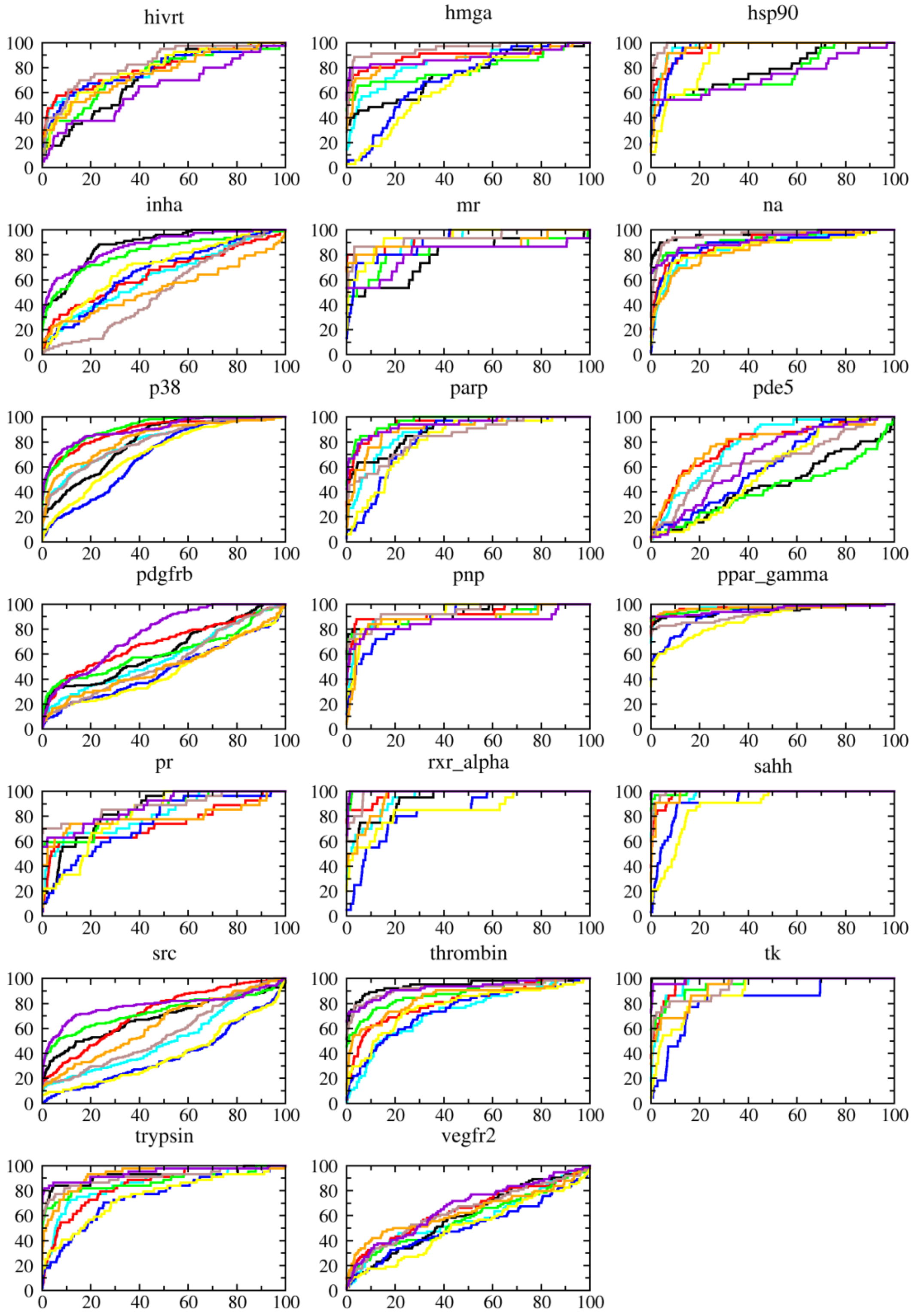

Figure S6. Receiver Operating Characteristic (ROC) curves for recovery of DUD actives from ZINC using various fingerprints. City block distance ($CBD_{fingerprint}$) was used as scoring function. 3DAPfp=Cyan; 3DXfp=Black; R3DAPfp=Red; R3DXfp=Green; PMIfp=Blue; USR=Yellow; USRCAT=Brown; APfp=Orange; Xfp=Violet. X-axis is % of sorted database and Y-axis=% of actives found.

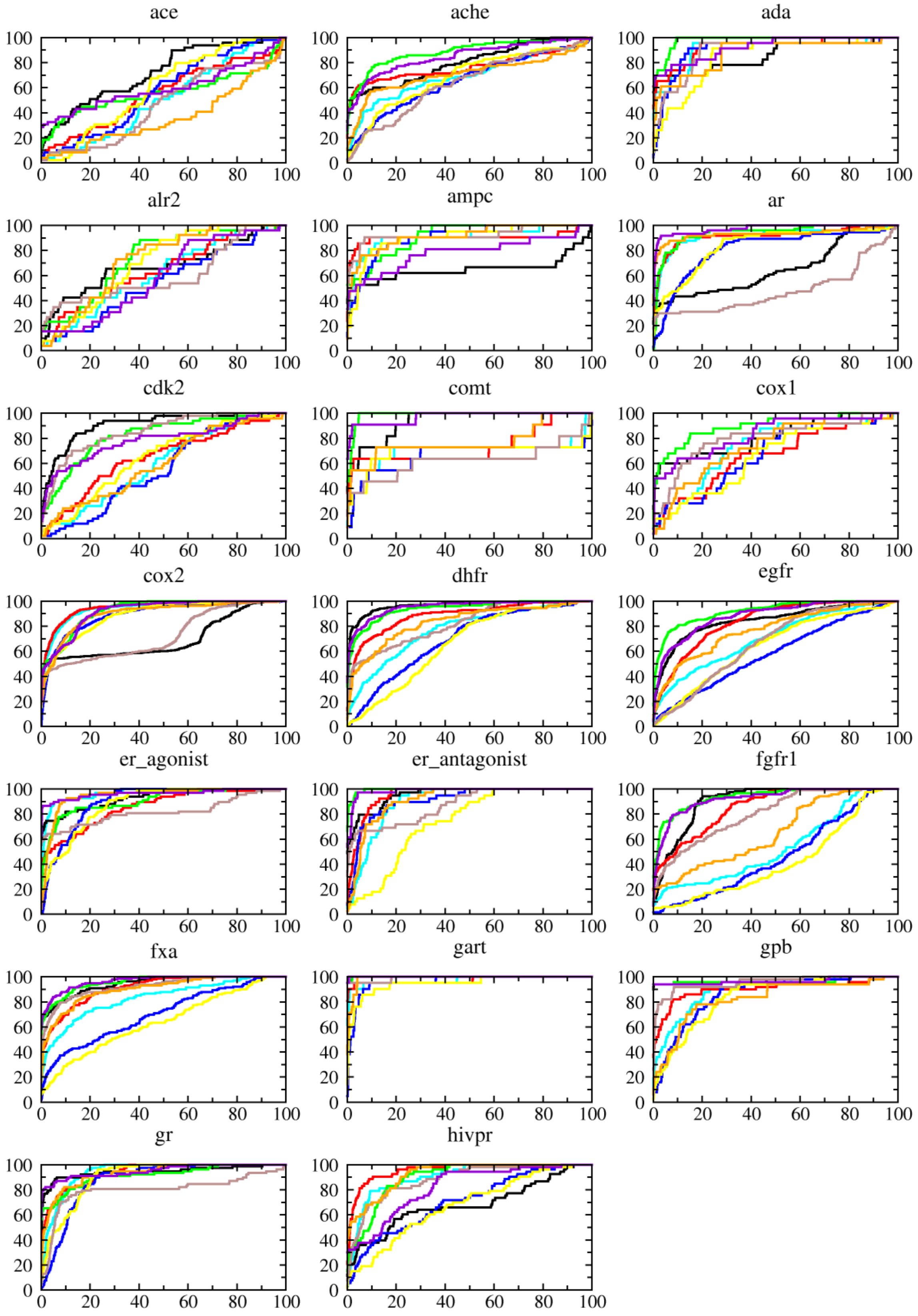
ace
ache
ada
alr2
ampc
ar
cdk2
comt
cox1
cox2
dhfr
egfr
er_agonist
er_antagonist
fgfr1
fxa
gart
gpb
gr
hivpr
0 20 40 60 80 100

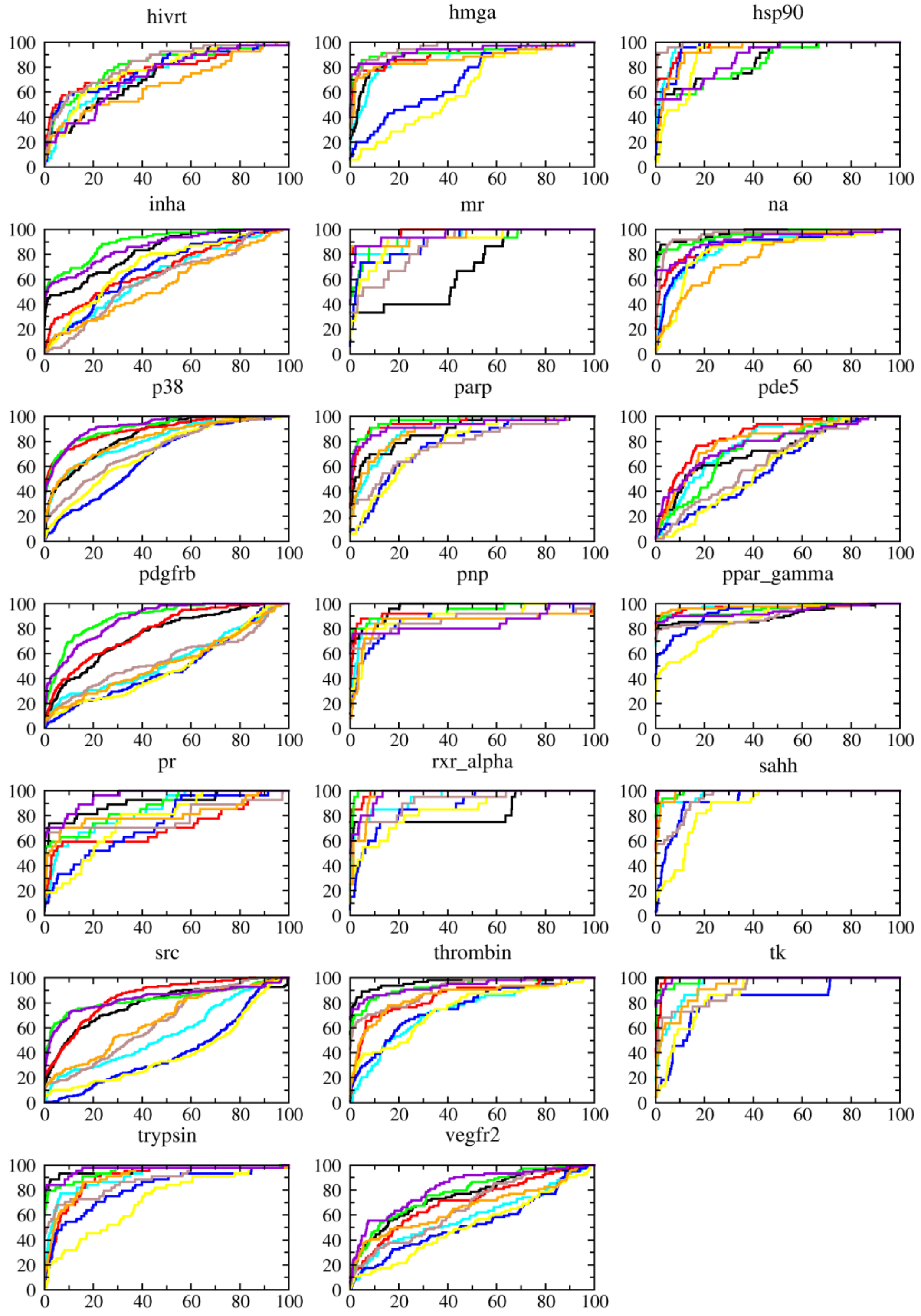

Figure S7. Receiver Operating Characteristic (ROC) curves for recovery of DUD actives from ZINC using various fingerprints. Tanimoto coefficient ($T_{\mathrm{fingerprint}}$) was used as scoring function. 3DAPfp=Cyan; 3DXfp=Black; R3DAPfp=Red; R3DXfp=Green; PMIfp=Blue; USR=Yellow; USRCAT=Brown; APfp=Orange; Xfp=Violet. X-axis is % of sorted database and Y-axis=% of actives found.

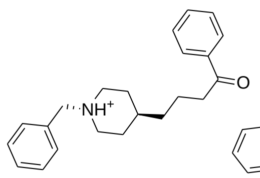

S1
ref. ache
act. + dec. = 3819 cpds

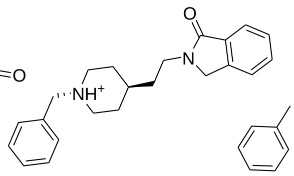

S2
rank = 2
CBD_{3Dxfp} = 57
T_{Sfp} = 0.52

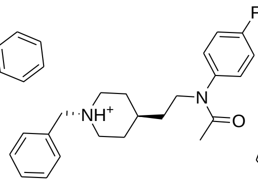

S3
rank = 13
CBD_{3DXfp} = 193
T_{Sfp} = 0.47

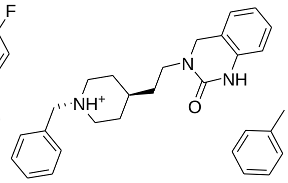

S4
rank = 32
CBD_{3DXfp} = 246
T_{Sfp} = 0.38

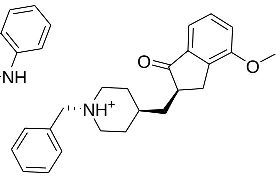

S5
rank = 51
CBD_{3DXfp} = 308
T_{Sfp} = 0.53

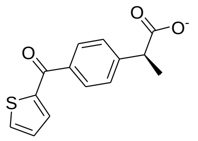

S6
ref. cox1
act. + dec. = 874 cpds

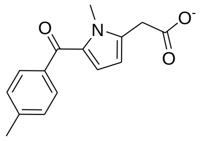

S7
rank = 5
CBD_{3DXfp} = 84
T_{Sfp} = 0.36

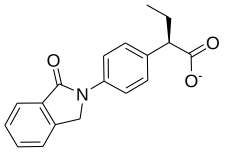

S8
rank = 10
CBD_{3DXfp} = 106
T_{Sfp} = 0.30

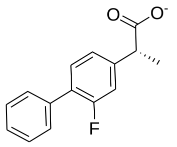

S9
rank = 36
CBD_{3DXfp} = 137
T_{Sfp} = 0.36

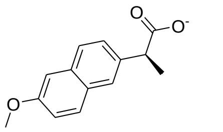

S10
rank = 85
CBD_{3DXfp} = 155
T_{Sfp} = 0.33

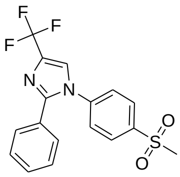

S11
ref. cox2
act. + dec. =12812 cpds

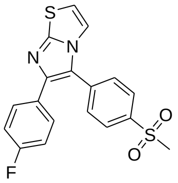

S12
rank = 18
CBD_{3DXfp} = 144
T_{Sfp} = 0.33

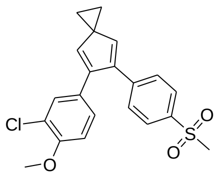

S13
rank = 31
CBD_{3DXfp} = 65
T_{Sfp} = 0.25

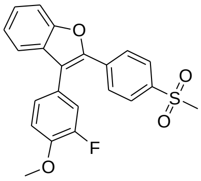

S14
rank = 47
CBD_{3DXfp} = 89
T_{Sfp} = 0.26

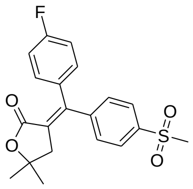

S15
rank = 50
CBD_{3DXfp} = 95
T_{Sfp} = 0.24

Figure S8. Examples of lead-hopping VS hits identified by LBVS of DUD actives from decoys using 3DXfp.

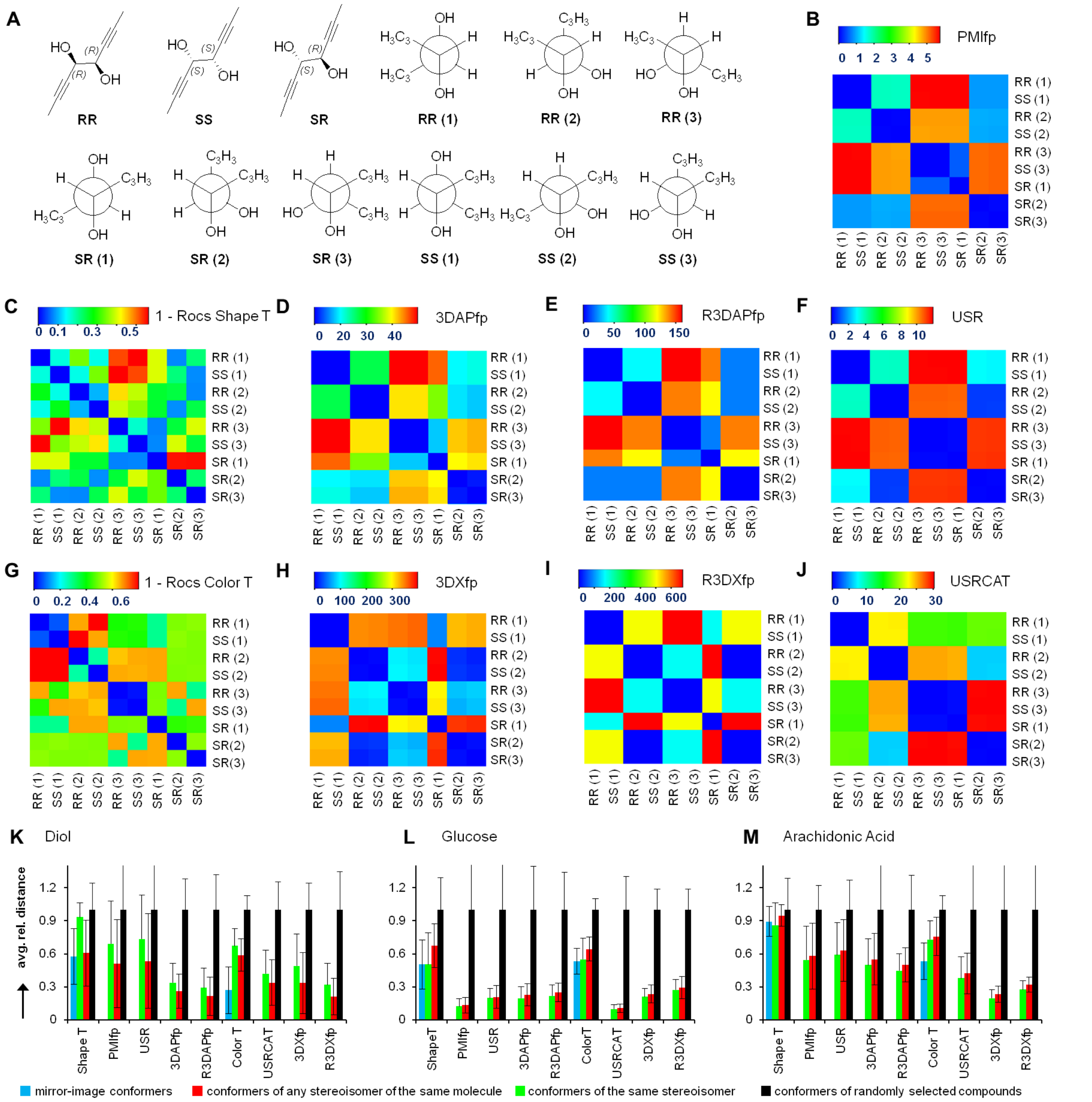

Figure S9. Stereoisomer and conformer comparisons. **A**. Stereoisomers and conformers of 4,5-dihydroxy-octa-2,6-diyne (Diol). **B-J**. Heatmaps showing pairwise distances between each conformer of 4,5-dihydroxy-octa-2,6-diyne. **K-M**. Average relative distance values and standard deviation (error bars) between exact mirror-image conformers (blue), conformers of the same stereoisomer (green), conformers of any stereoisomer of the same molecule (red), compared to average relative distances between conformers of randomly selected molecules of same size (HAC) from CSD (black), for 4,5-dihydroxy-octa-2,6-diyne (diol, 9 conformers, K), glucopyranose (glucose, 154 conformers, L), and (5Z,8Z,11Z,14Z)-5,8,11,14-eicosatetraenoic acid (arachidonic acid, 640 conformers, M). Conformers were generated by Omega.

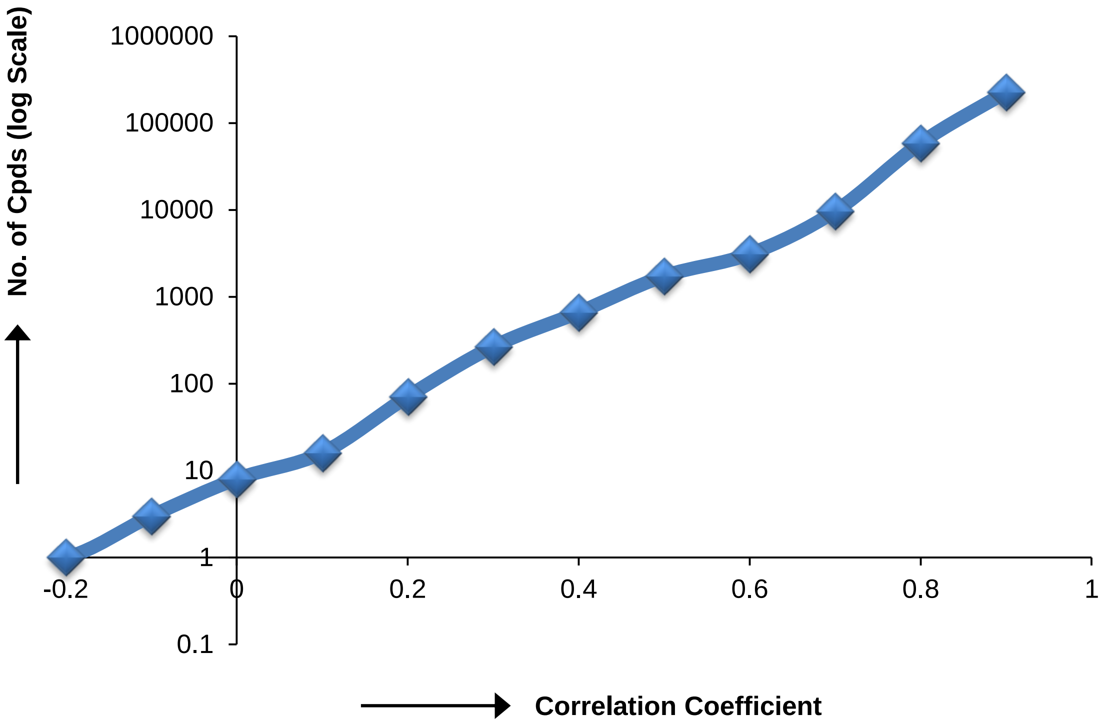

Figure S10. Pearson correlation coefficient between through-space and topological distances for ~325k small molecules extracted from PDB database. For each PDB compound, euclidian distance and topological distance were calculated for all possible atom-pairs within a molecule (atom pairs with topological distance of ≤ 3 were excluded), which were then used for calculation of correlation coefficient. The 1000 molecules with the lowest correlation were surveyed and found to contain many glycerol and lipid conformers. The 10 examples chosen in Figure S11are cases of drug-protein complexes.

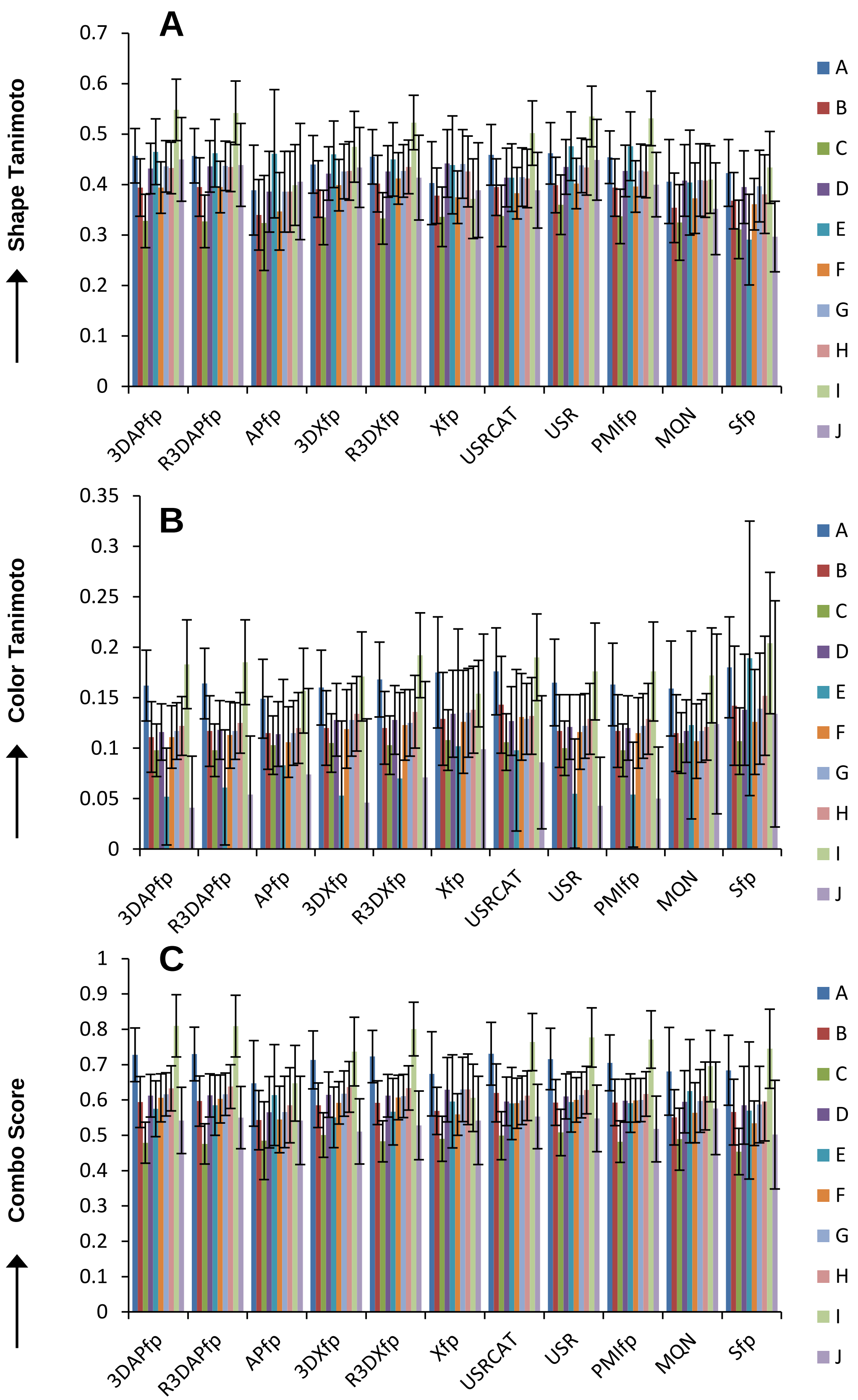

Figure S11. Average ROCS similarity scores A) Shape Tanimoto B) Color Tanimoto and C) Combo Score for 10,000 nearest neighbors of 10 folded compounds (A-J) retrieved from ZINC database using various fingerprints spaces. Folded compounds were obtained from PDB (codes: 4jfm, 3u8h, 3t8v, 4ps5, 1adl, 1kvo, 4ogj, 4o73, 2ybu, 1cvu).
